# Supplementary figures and images for: TIGAR deficiency enhances skeletal muscle thermogenesis by increasing neuromuscular junction cholinergic signaling
Source: eLife. 2022 Mar 7;11:e73360. doi: 10.7554/eLife.73360 (PMC8947760; doi:10.7554/eLife.73360)

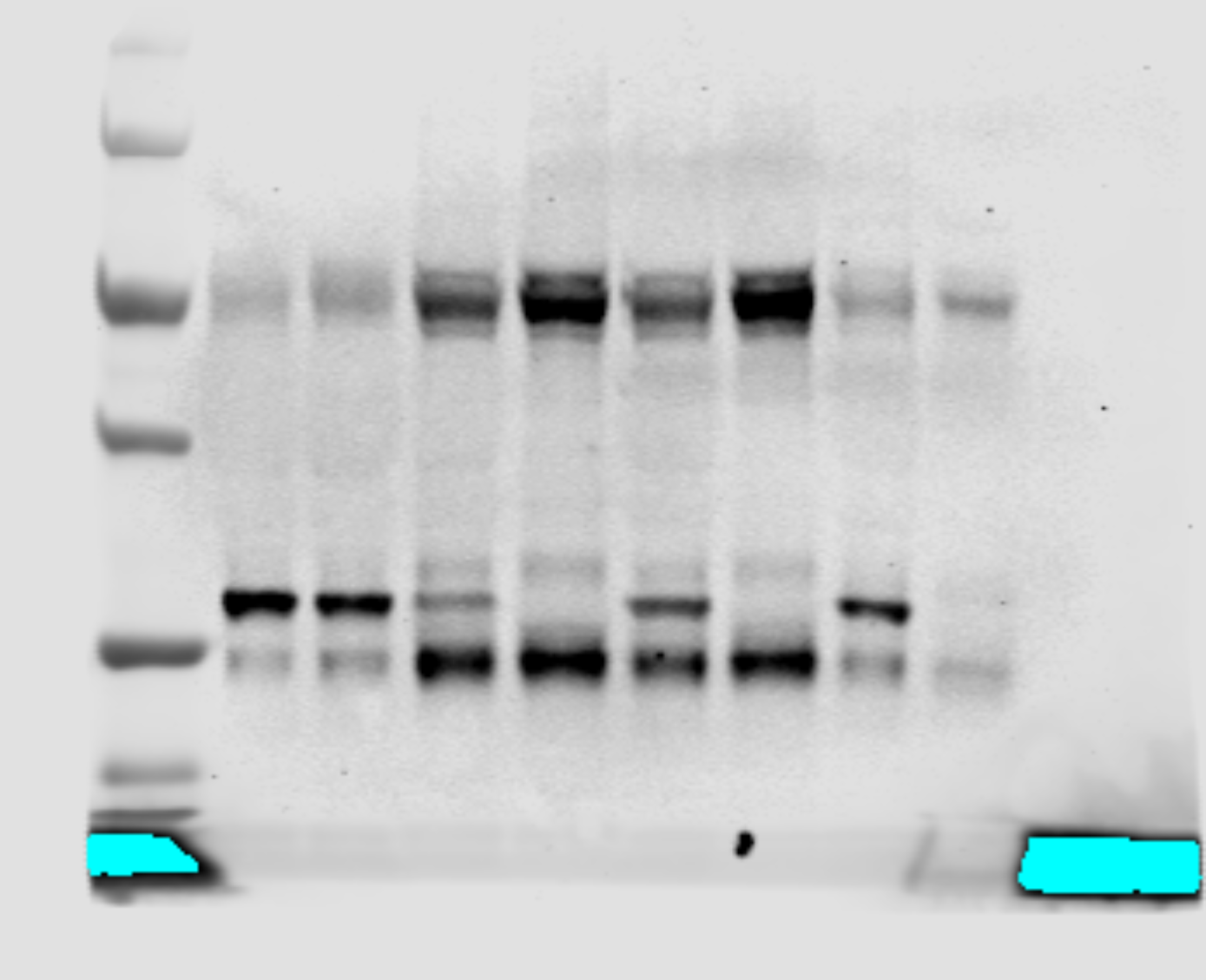

Supplement: Figure 1—source data 1. — The raw image was used for Figure 1C. A detailed description of the raw images is shown in Source data 1. [file elife-73360-fig1-data1.zip › Tang_25-08-2021-RA-eLife-73360R1_Figure_1_source_data_1.png]

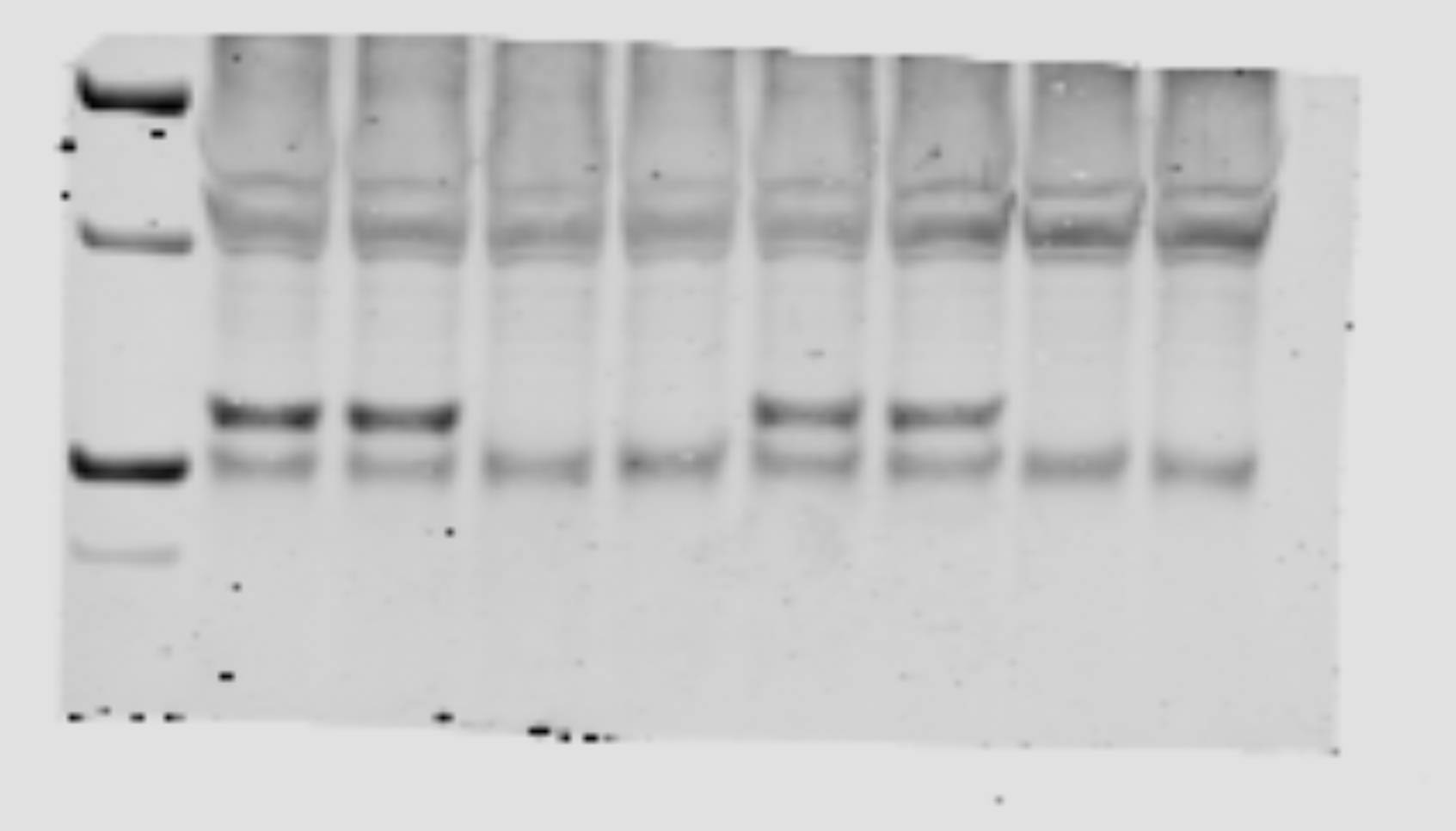

Supplement: Figure 1—source data 2. — The raw image was used for Figure 1E. A detailed description of the raw images is shown in Source data 1. [file elife-73360-fig1-data2.zip › Tang_25-08-2021-RA-eLife-73360R1_Figure_1_source_data_2.png]

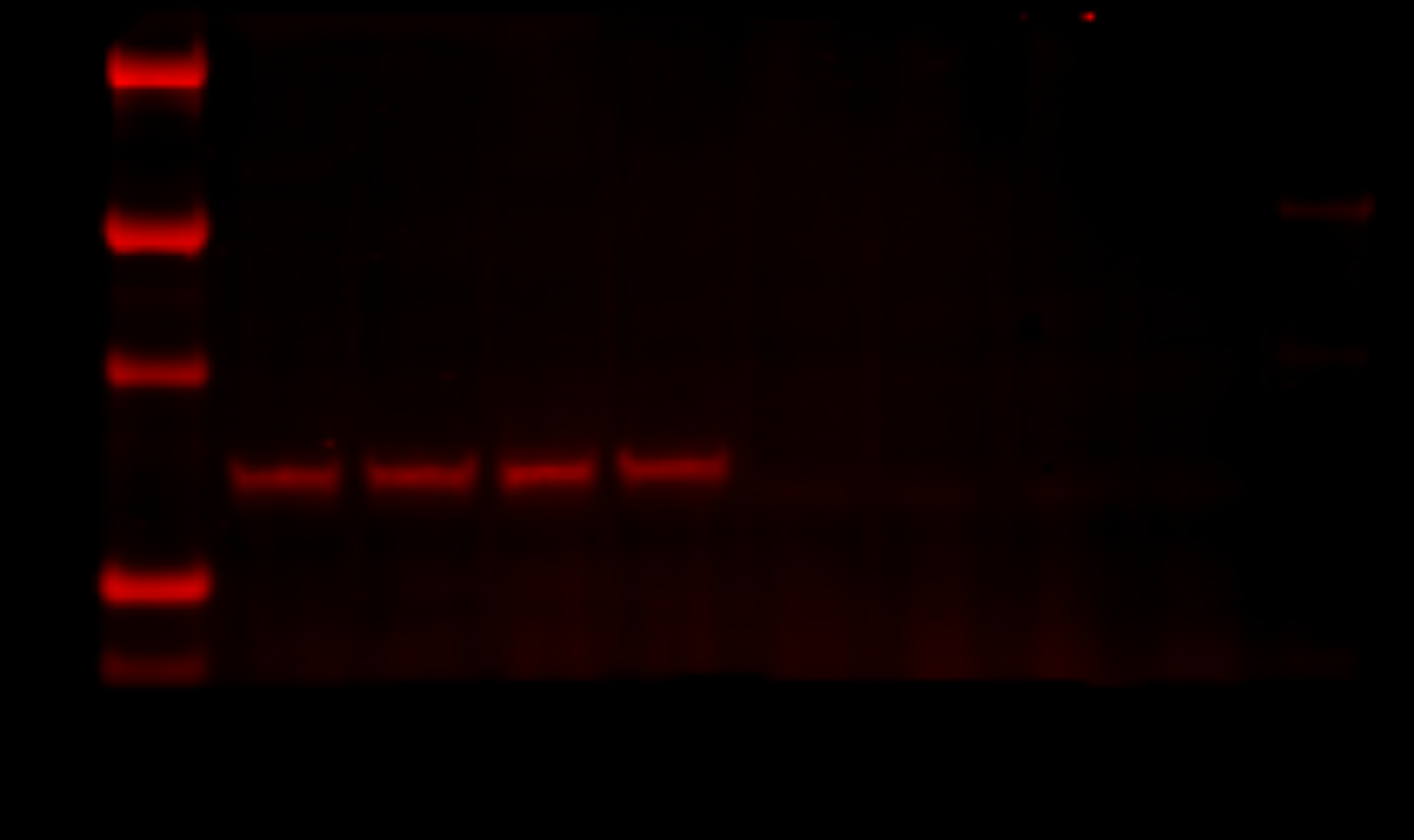

Supplement: Figure 1—source data 3. — The raw image was used for Figure 1E. A detailed description of the raw images is shown in Source data 1. [file elife-73360-fig1-data3.zip › Tang_25-08-2021-RA-eLife-73360R1_Figure_1_source_data_3.png]

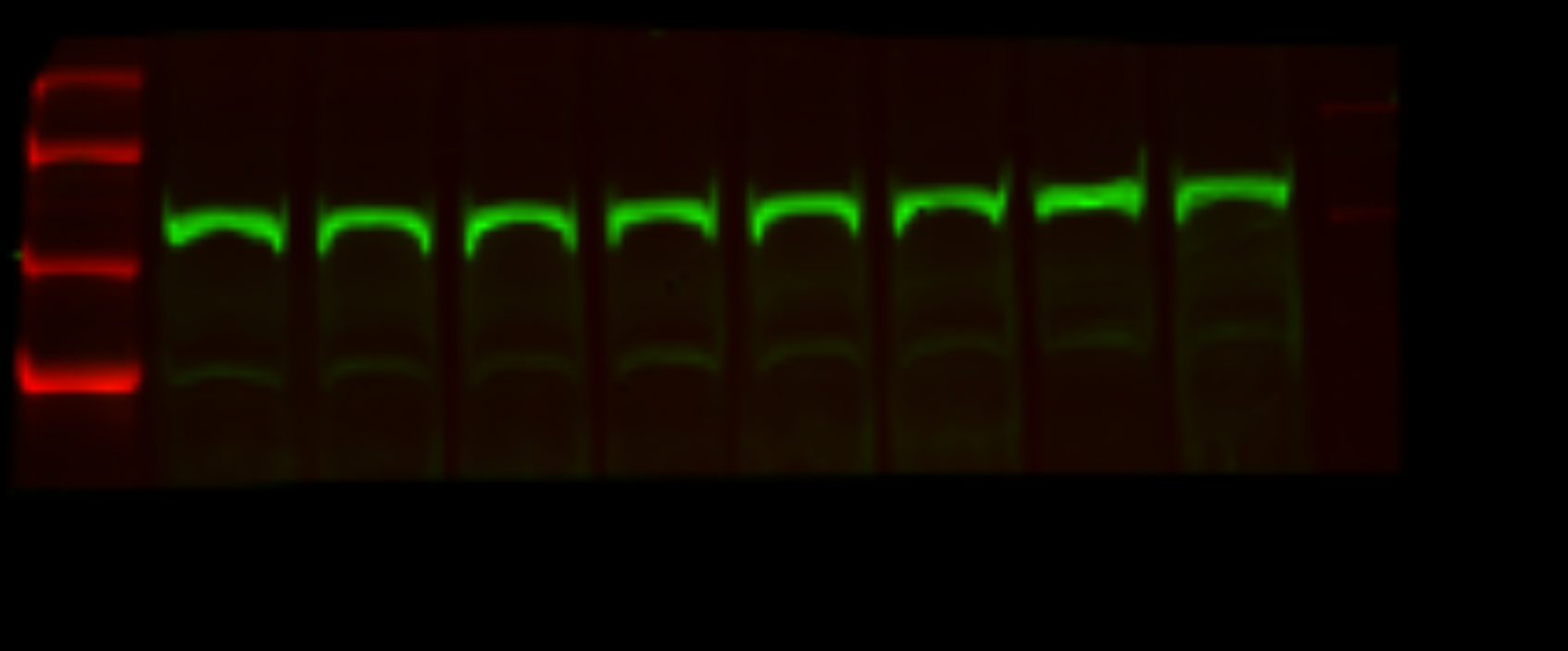

Supplement: Figure 1—source data 4. — The raw image was used for Figure 1E. A detailed description of the raw images is shown in Source data 1. [file elife-73360-fig1-data4.zip › Tang_25-08-2021-RA-eLife-73360R1_Figure_1_source_data_4.png]

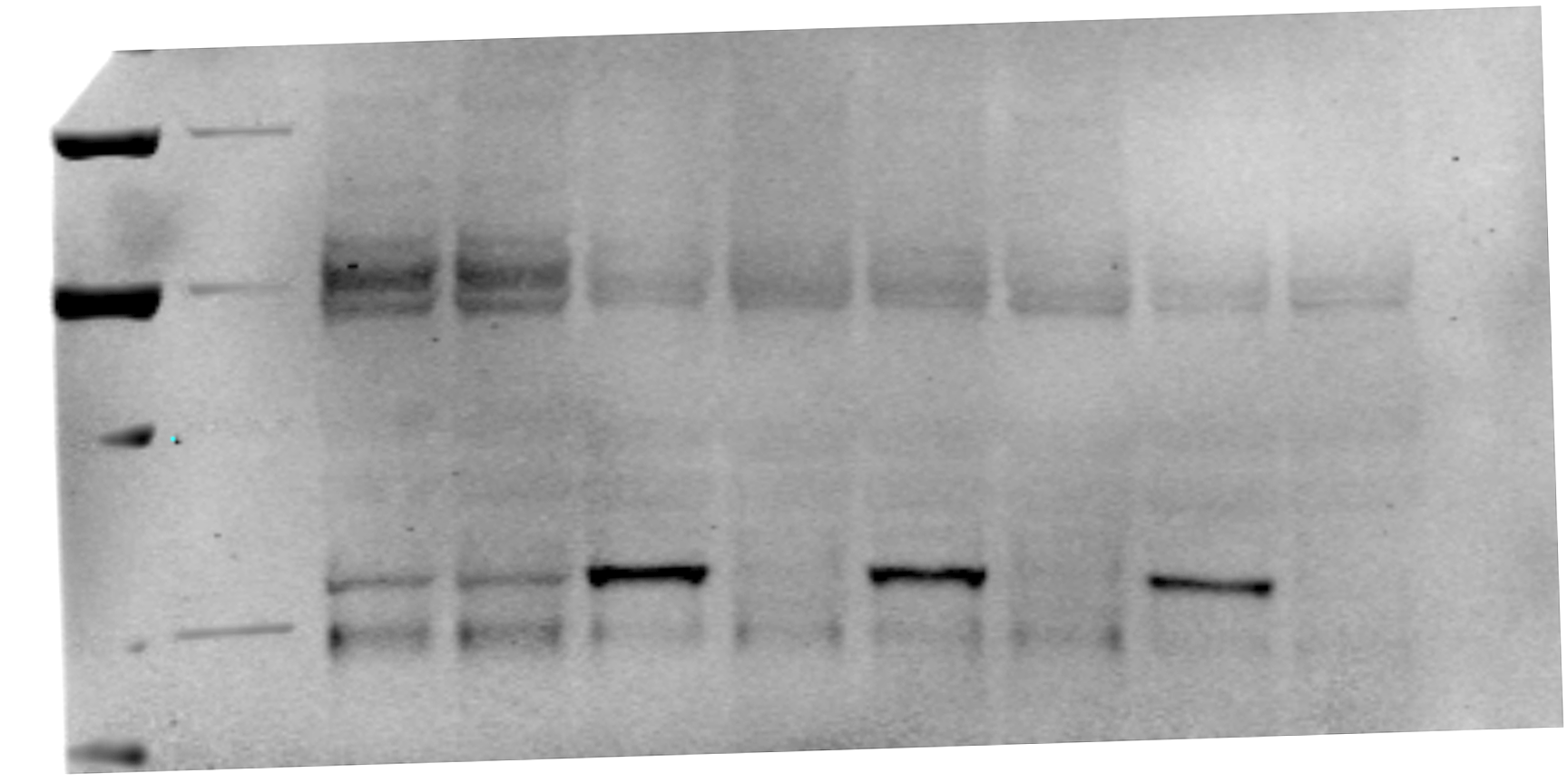

Supplement: Figure 1—source data 5. — The raw image was used for Figure 1G. A detailed description of the raw images is shown in Source data 1. [file elife-73360-fig1-data5.zip › Tang_25-08-2021-RA-eLife-73360R1_Figure_1_source_data_5.png]

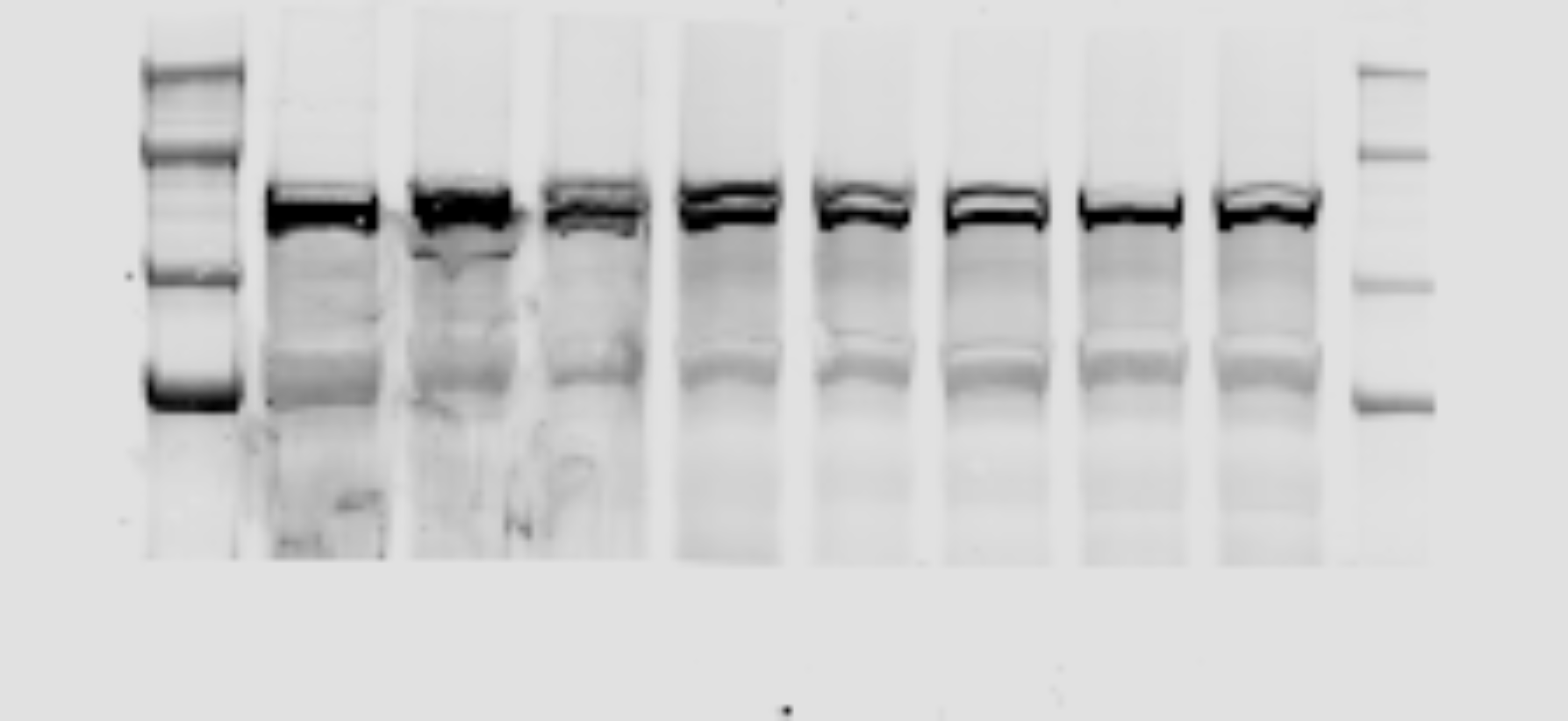

Supplement: Figure 1—source data 6. — The raw image was used for Figure 1G. A detailed description of the raw images is shown in Source data 1. [file elife-73360-fig1-data6.zip › Tang_25-08-2021-RA-eLife-73360R1_Figure_1_source_data_6.png]

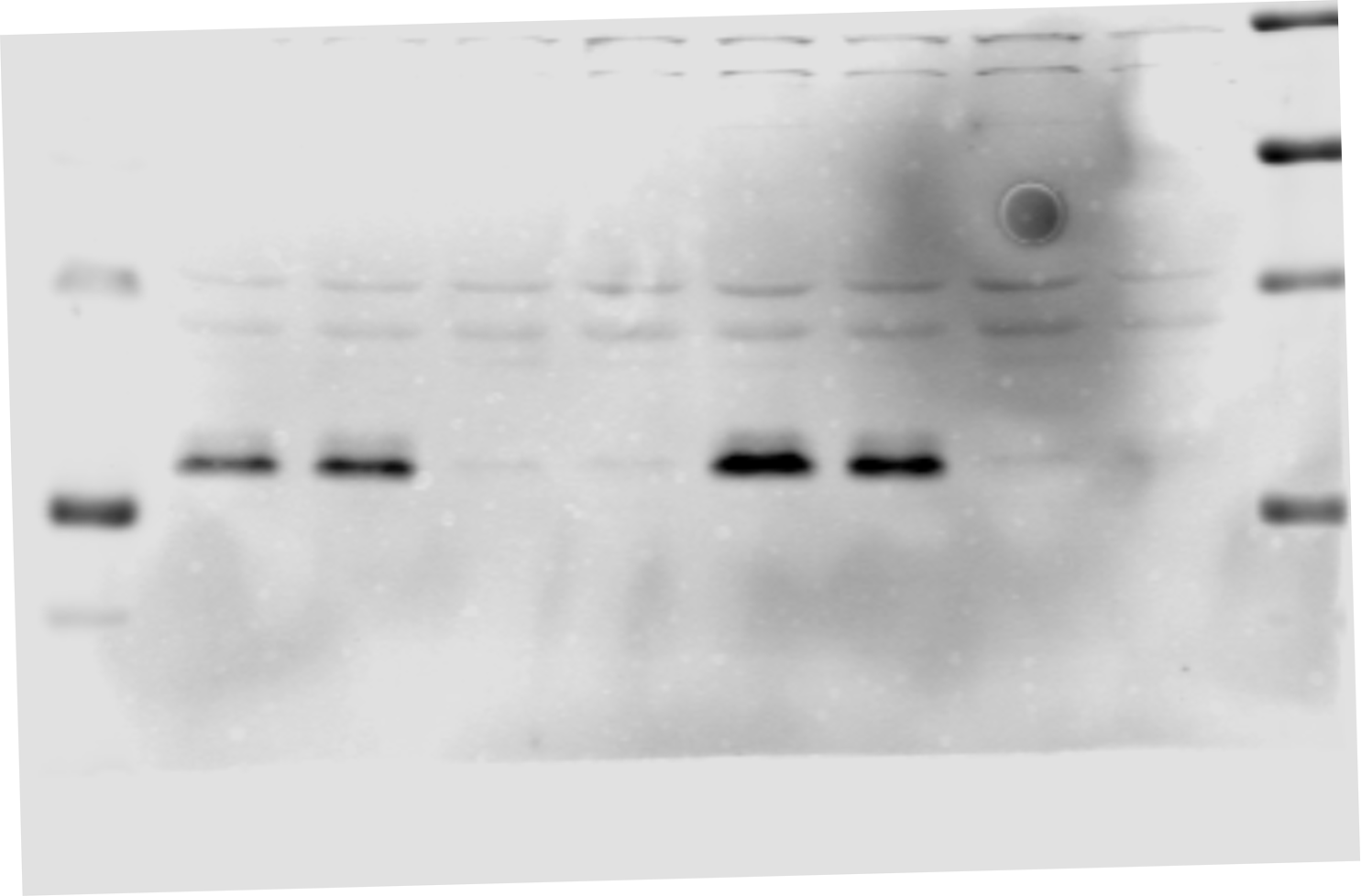

Supplement: Figure 1—figure supplement 1—source data 1. — The raw image was used for Figure 1—figure supplement 1B. A detailed description of the raw images is shown in Source data 1. [file elife-73360-fig1-figsupp1-data1.zip › Tang_25-08-2021-RA-eLife-73360R1_Figure_1_figure_supplement_1_source_data_1.png]

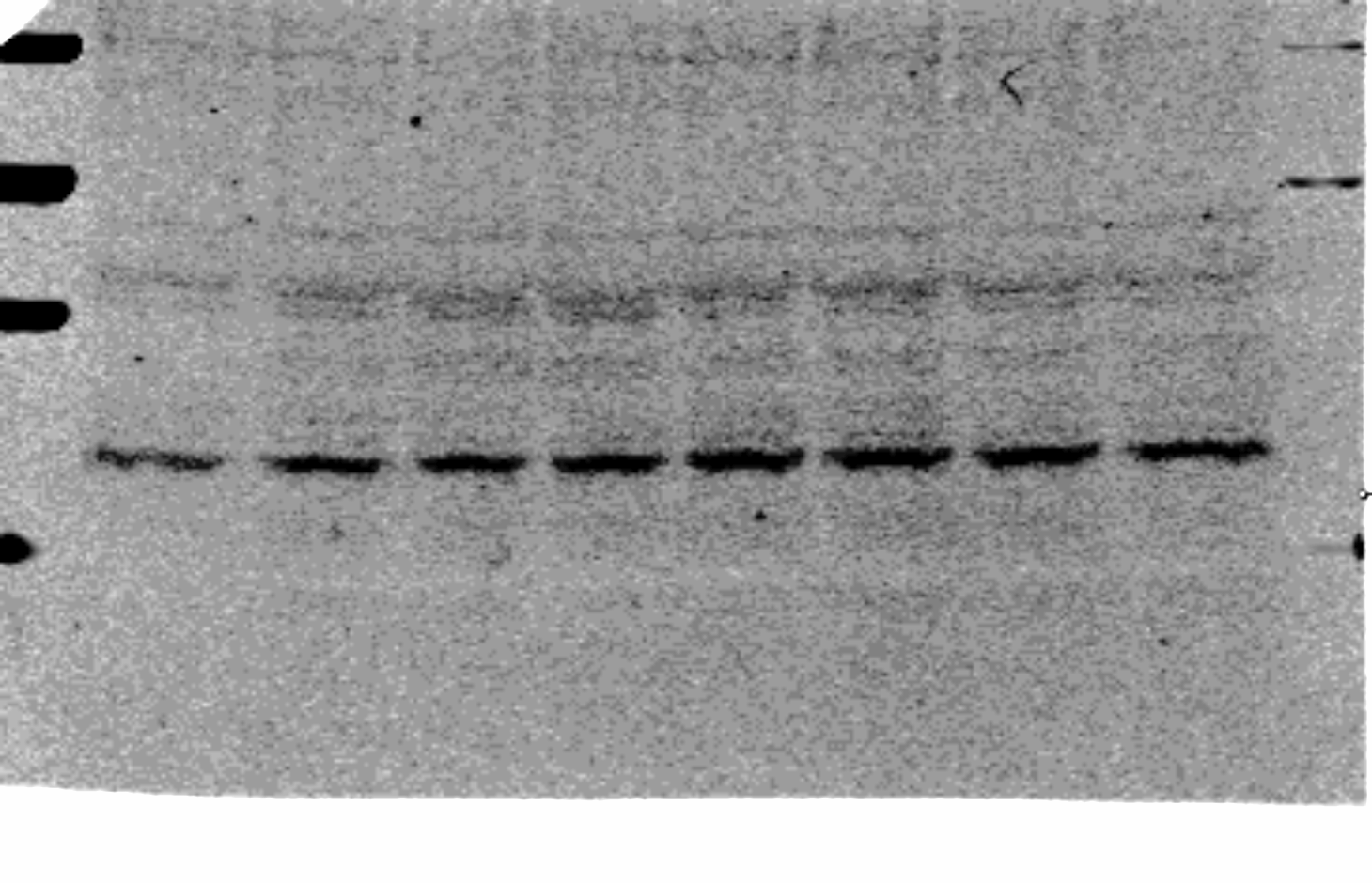

Supplement: Figure 1—figure supplement 1—source data 2. — The raw image was used for Figure 1—figure supplement 1B. A detailed description of the raw images is shown in Source data 1. [file elife-73360-fig1-figsupp1-data2.zip › Tang_25-08-2021-RA-eLife-73360R1_Figure_1_figure_supplement_1_source_data_2.png]

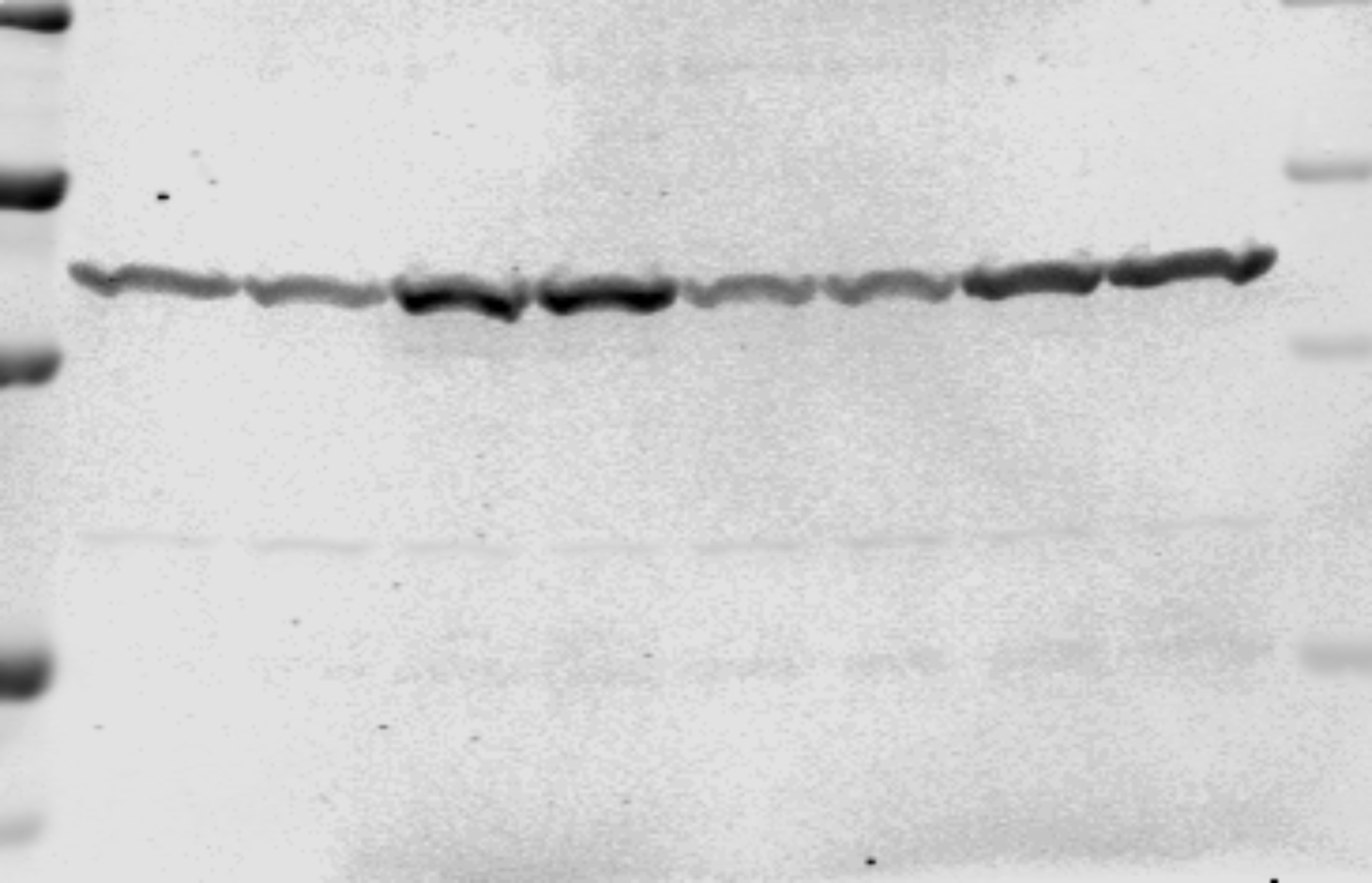

Supplement: Figure 1—figure supplement 1—source data 3. — The raw image was used for Figure 1—figure supplement 1B. A detailed description of the raw images is shown in Source data 1. [file elife-73360-fig1-figsupp1-data3.zip › Tang_25-08-2021-RA-eLife-73360R1_Figure_1_figure_supplement_1_source_data_3.png]

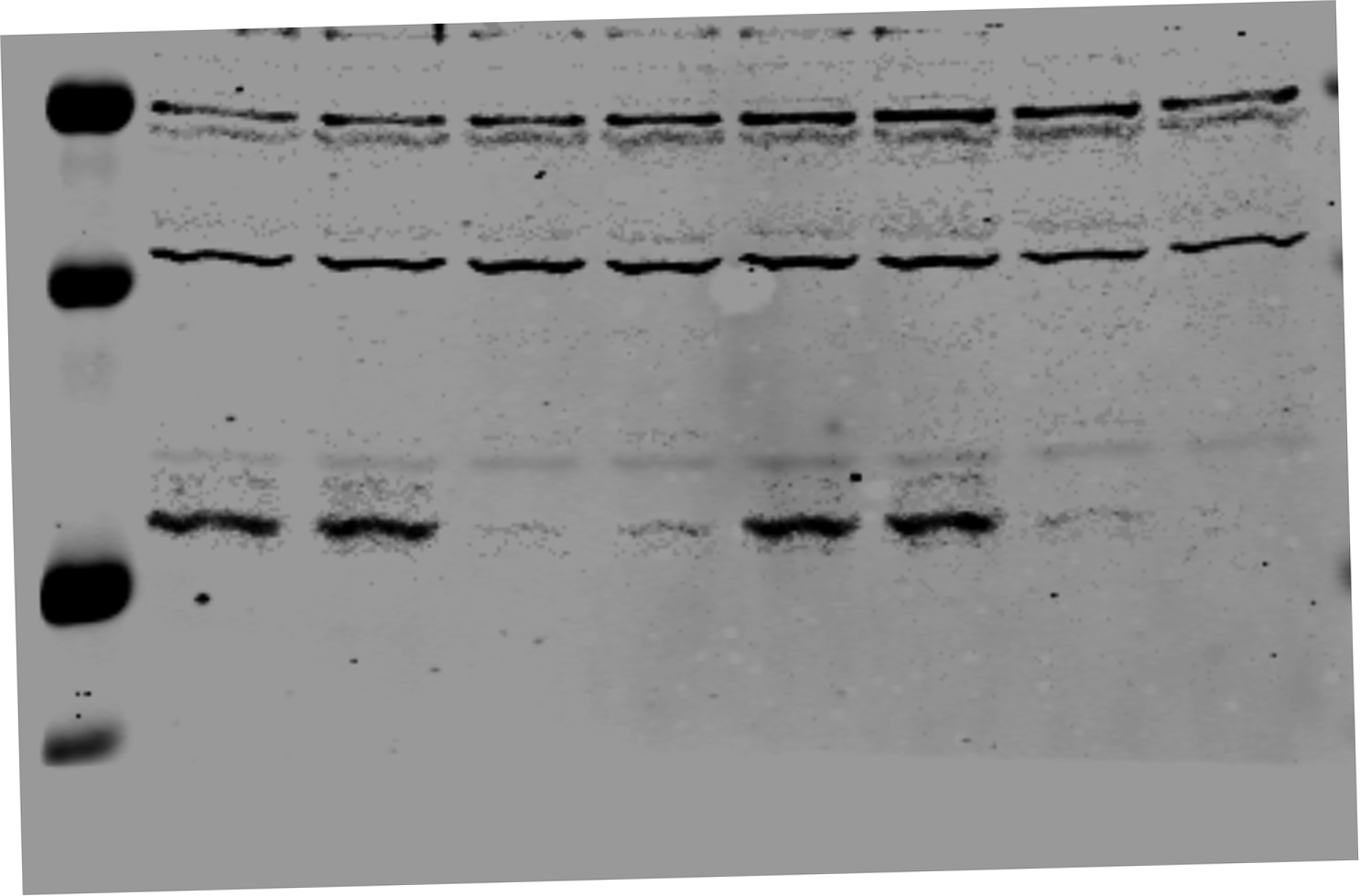

Supplement: Figure 4—source data 1. — The SCGs from the three mice of the same strain were pooled and processed by beads homogenization to acquire tissue lysates. 15 μg of the tissue lysate were used for TIGAR (30 kDa) immunoblotting analysis as described in the ‘Immunoblotting’ section. The left four lanes of the raw image (lysate from the mice at ambient temperature) were used for Figure 4B to confirm the efficiency of TIGAR protein loss in SCG of the chTKO mice. A detailed description of the raw images is shown in Source data 1. [file elife-73360-fig4-data1.zip › Tang_25-08-2021-RA-eLife-73360R1_Figure_4_source_data_1.png]

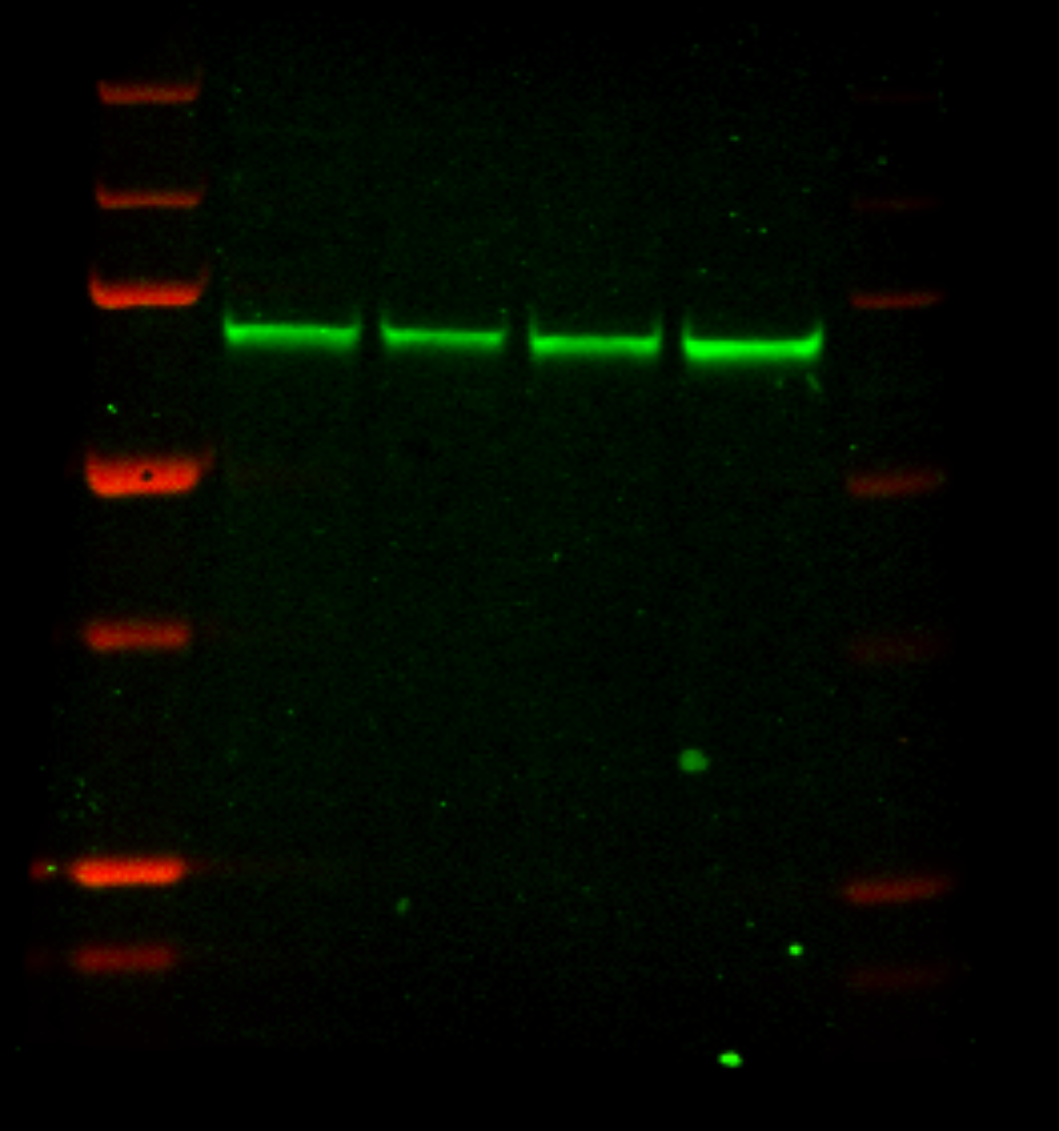

Supplement: Figure 4—source data 2. — The SCGs from the three mice of the same strain were pooled and processed by beads homogenization to acquire tissue lysates. 15 μg of the tissue lysate were used for choline acetyltransferase (ChAT, 70 kDa) immunoblotting analysis as described in the ‘Immunoblotting’ section. The left four lanes of the raw image (lysate from the mice at ambient temperature) were used for Figure 4B. A detailed description of the raw images is shown in Source data 1. [file elife-73360-fig4-data2.zip › Tang_25-08-2021-RA-eLife-73360R1_Figure_4_source_data_2.png]

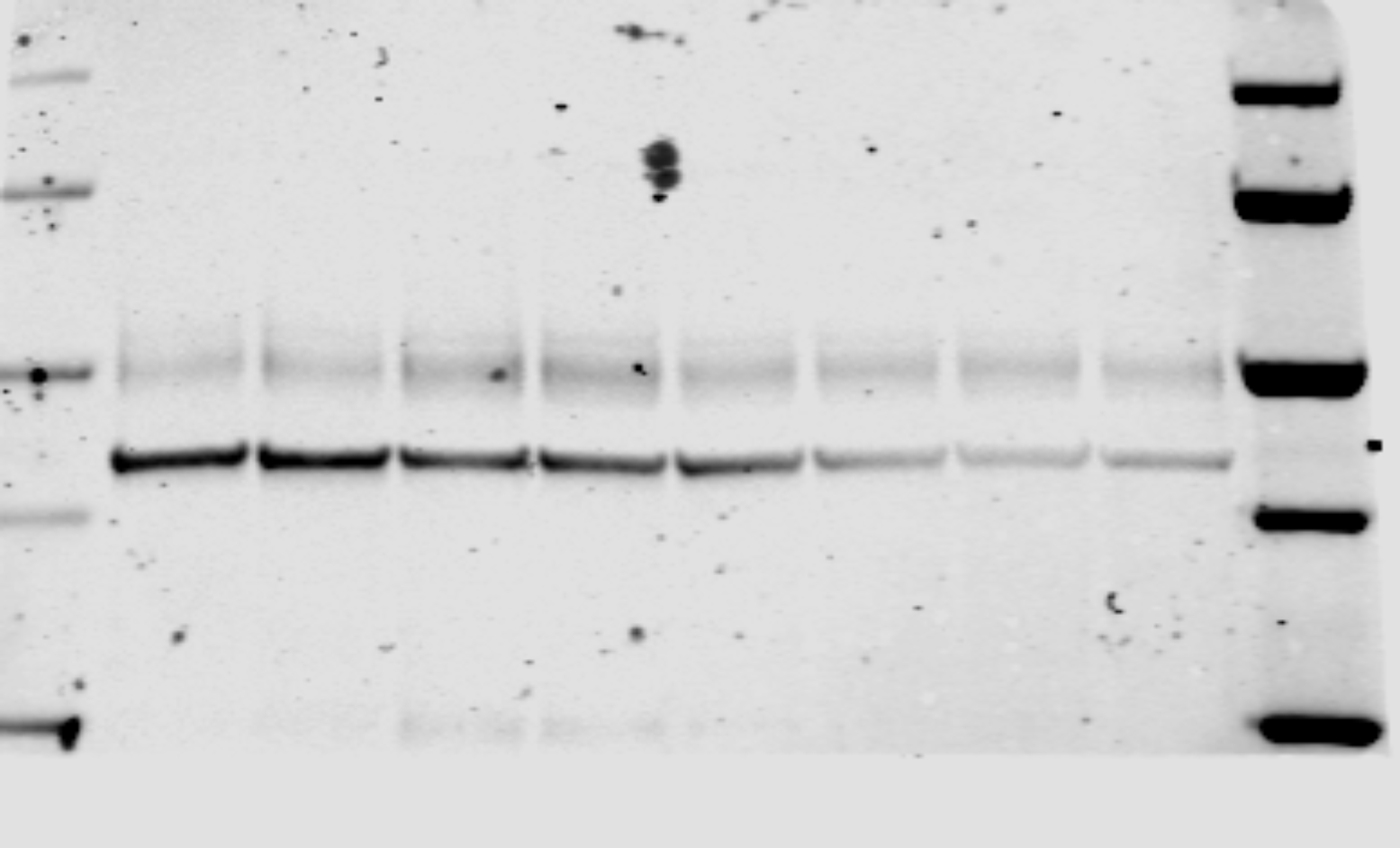

Supplement: Figure 4—source data 3. — The SCGs from the three mice of the same strain were pooled and processed by beads homogenization to acquire tissue lysates. 15 μg of the tissue lysate were used for actin β (42 kDa) immunoblotting analysis as described in the ‘Immunoblotting’ section. The left four lanes of the raw image (lysate from the mice at ambient temperature) were used for Figure 4B. A detailed description of the raw images is shown in Source data 1. [file elife-73360-fig4-data3.zip › Tang_25-08-2021-RA-eLife-73360R1_Figure_4_source_data_3.png]

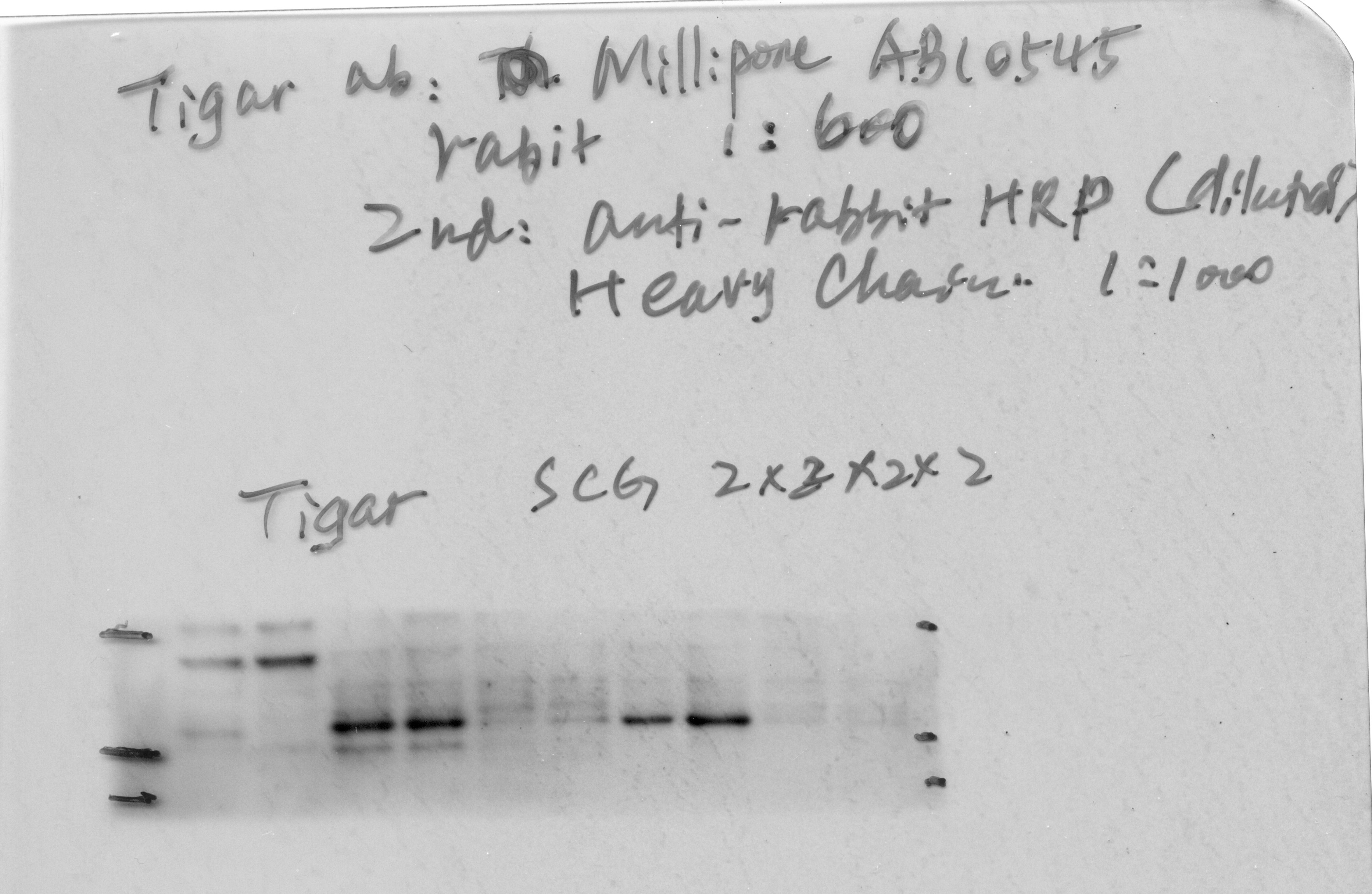

Supplement: Figure 4—source data 4. — The SCGs from the three mice of the same strain were pooled and processed by beads homogenization to acquire tissue lysates. 15 μg of the tissue lysate were used for TIGAR (30 kDa) immunoblotting analysis as described in the ‘Immunoblotting’ section. The right eight lanes of the raw image (lysate from SCG tissues) was used for Figure 4H to confirm the efficiency of TIGAR protein loss in SCG of the chTKO mice. The left two lanes of the raw immunoblotting image represent the lysates of soluble fraction (RIPA lysis buffer extracted) of whole-brain tissues from the ChatCre and chTKO mice, respectively. A detailed description of the raw images is shown in Source data 1. [file elife-73360-fig4-data4.zip › Tang_25-08-2021-RA-eLife-73360R1_Figure_4_source_data_4.png]

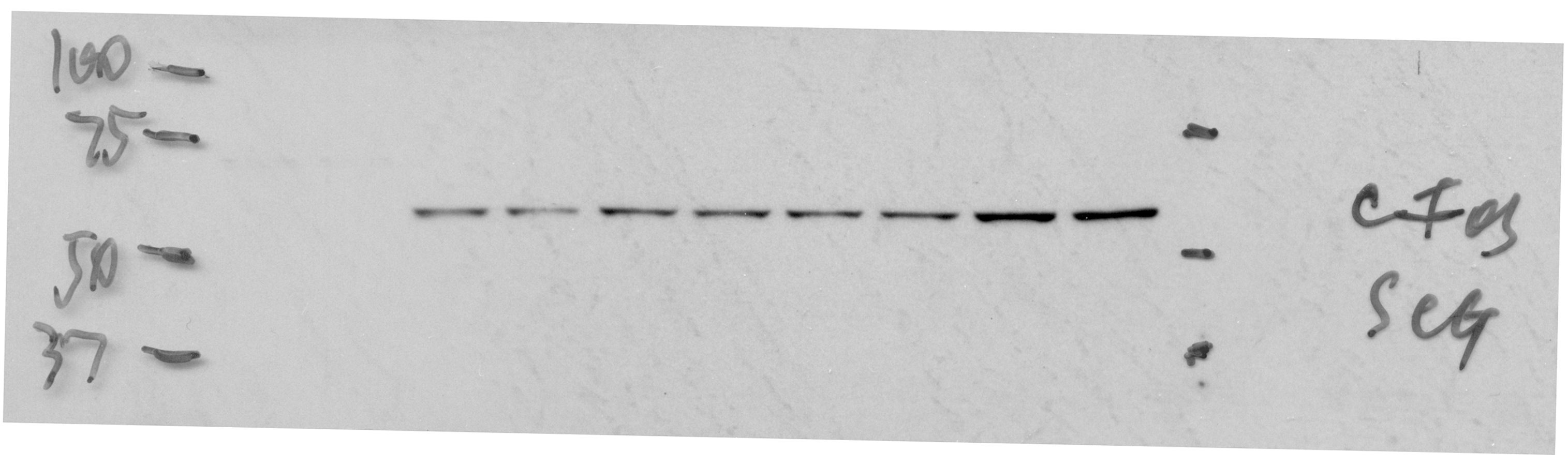

Supplement: Figure 4—source data 5. — The SCGs from the three mice of the same strain were pooled and processed by beads homogenization to acquire tissue lysates. 15 μg of the tissue lysate were used for c-Fos (62 kDa) immunoblotting analysis as described in the ‘Immunoblotting’ section. The right eight lanes of the raw image (lysate from SCG tissues) were used for Figure 4H to show the increase in c-Fos protein in SCG of the chTKO mice under cold exposed. The left two lanes of the raw immunoblotting image represent the lysates of soluble fraction (RIPA lysis buffer extracted) of whole-brain tissues from the ChatCre and chTKO mice, respectively. A detailed description of the raw images is shown in Source data 1. [file elife-73360-fig4-data5.zip › Tang_25-08-2021-RA-eLife-73360R1_Figure_4_source_data_5.png]

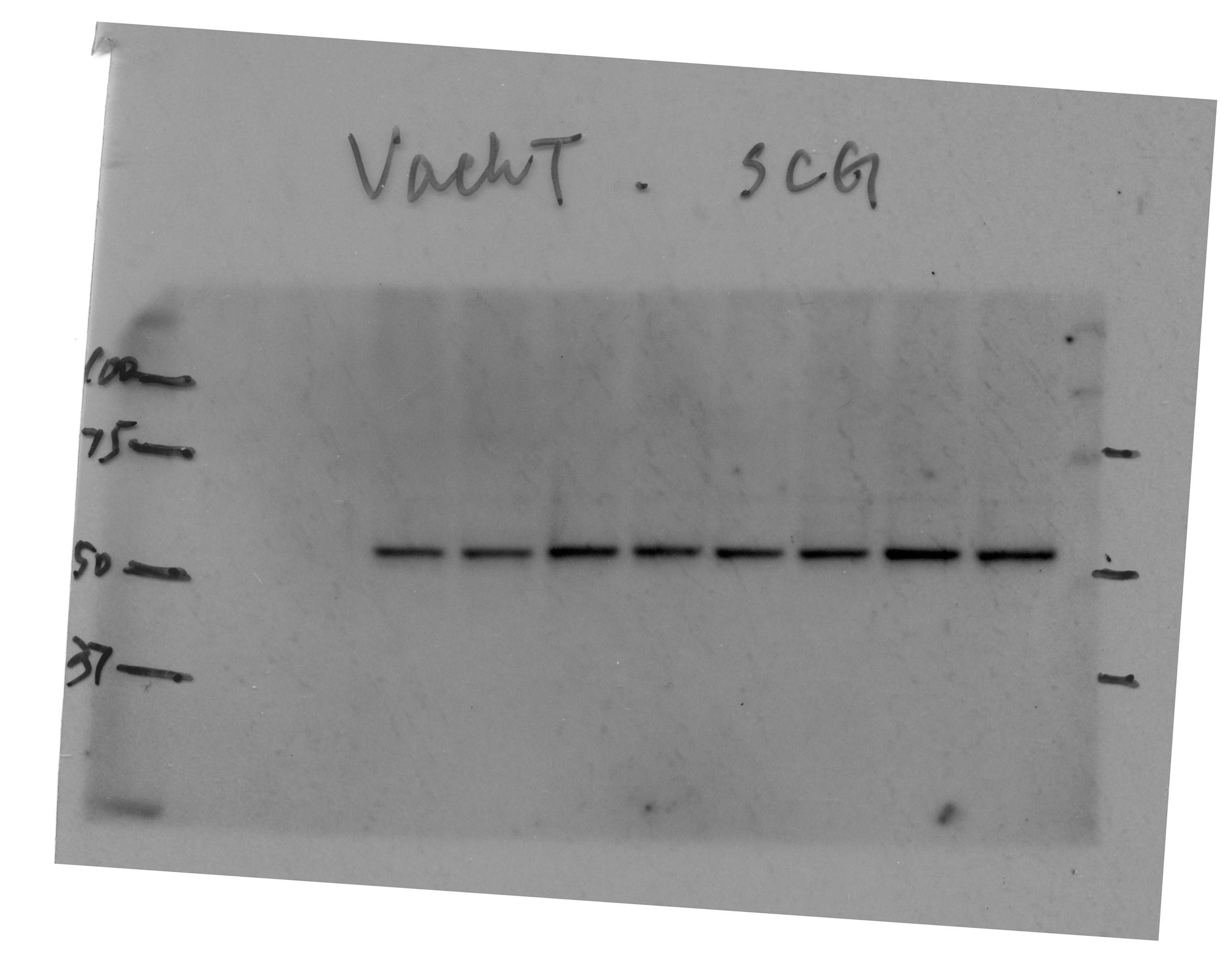

Supplement: Figure 4—source data 6. — The SCGs from the three mice of the same strain were pooled and processed by beads homogenization to acquire tissue lysates. 15 μg of the tissue lysate were used for vesicular acetylcholine transporter (VAChT, 55 kDa) immunoblotting analysis as described in the ‘Immunoblotting’ section. The right eight lanes of the raw image (lysate from SCG tissues) were used for Figure 4H to show VAChT protein in the SCGs. The left two lanes of the raw immunoblotting image represent the lysates of soluble fraction (RIPA lysis buffer extracted) of whole-brain tissues from the ChatCre and chTKO mice, respectively. A detailed description of the raw images is shown in Source data 1. [file elife-73360-fig4-data6.zip › Tang_25-08-2021-RA-eLife-73360R1_Figure_4_source_data_6.png]

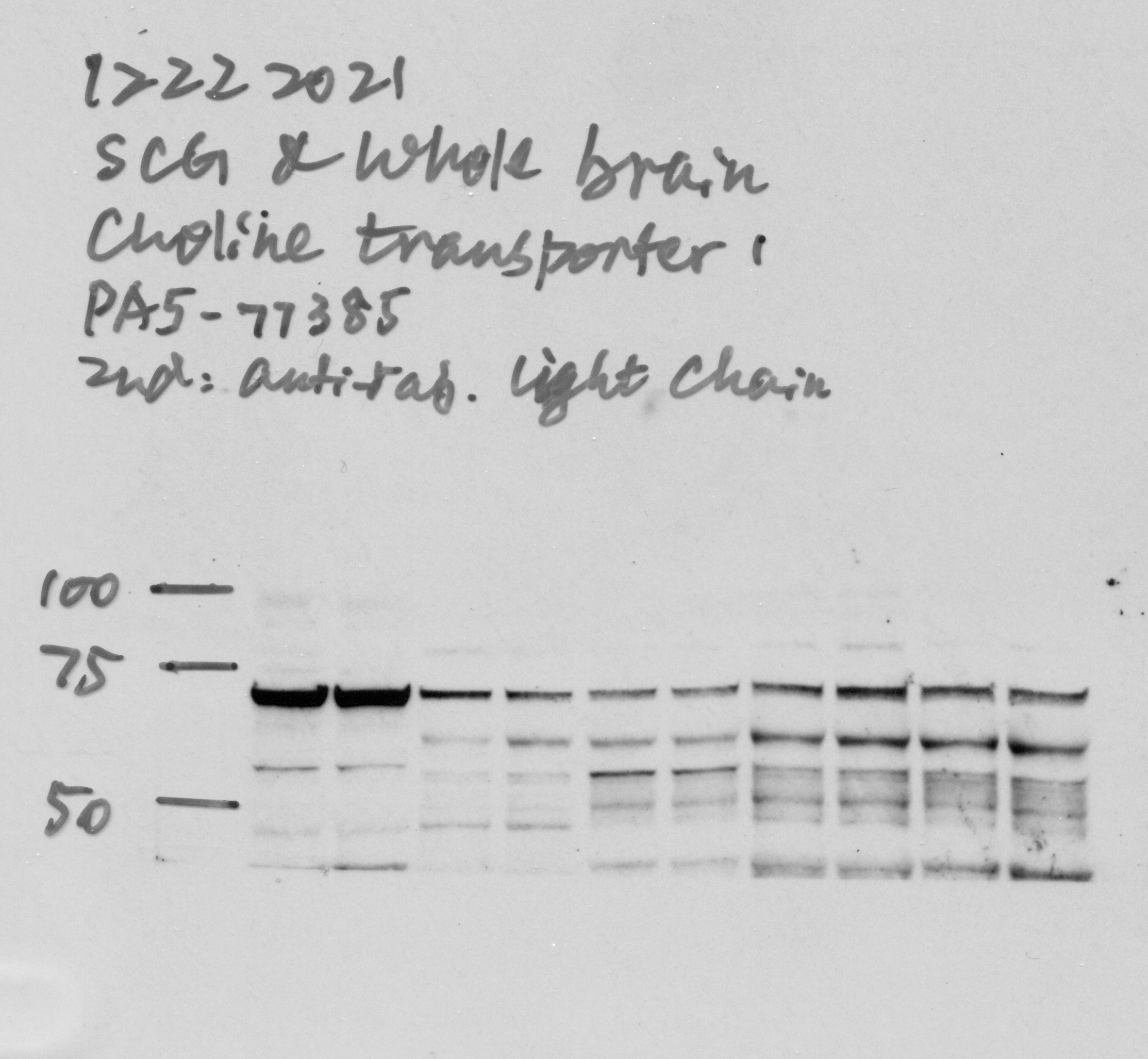

Supplement: Figure 4—source data 7. — The SCGs from the three mice of the same strain were pooled and processed by beads homogenization to acquire tissue lysates. 15 μg of the tissue lysate were used for choline transporter (ChT, 55 kDa) immunoblotting analysis as described in the ‘Immunoblotting’ section. The right eight lanes of the raw image (lysate from SCG tissues) were used for Figure 4H to show ChT protein in that 63 and 70 kDa bands were observed in the SCGs. The left two lanes of the raw immunoblotting image represent the lysates of soluble fraction (RIPA lysis buffer extracted) of whole-brain tissues from the ChatCre and chTKO mice, respectively. A detailed description of the raw images is shown in Source data 1. [file elife-73360-fig4-data7.zip › Tang_25-08-2021-RA-eLife-73360R1_Figure_4_source_data_7.png]

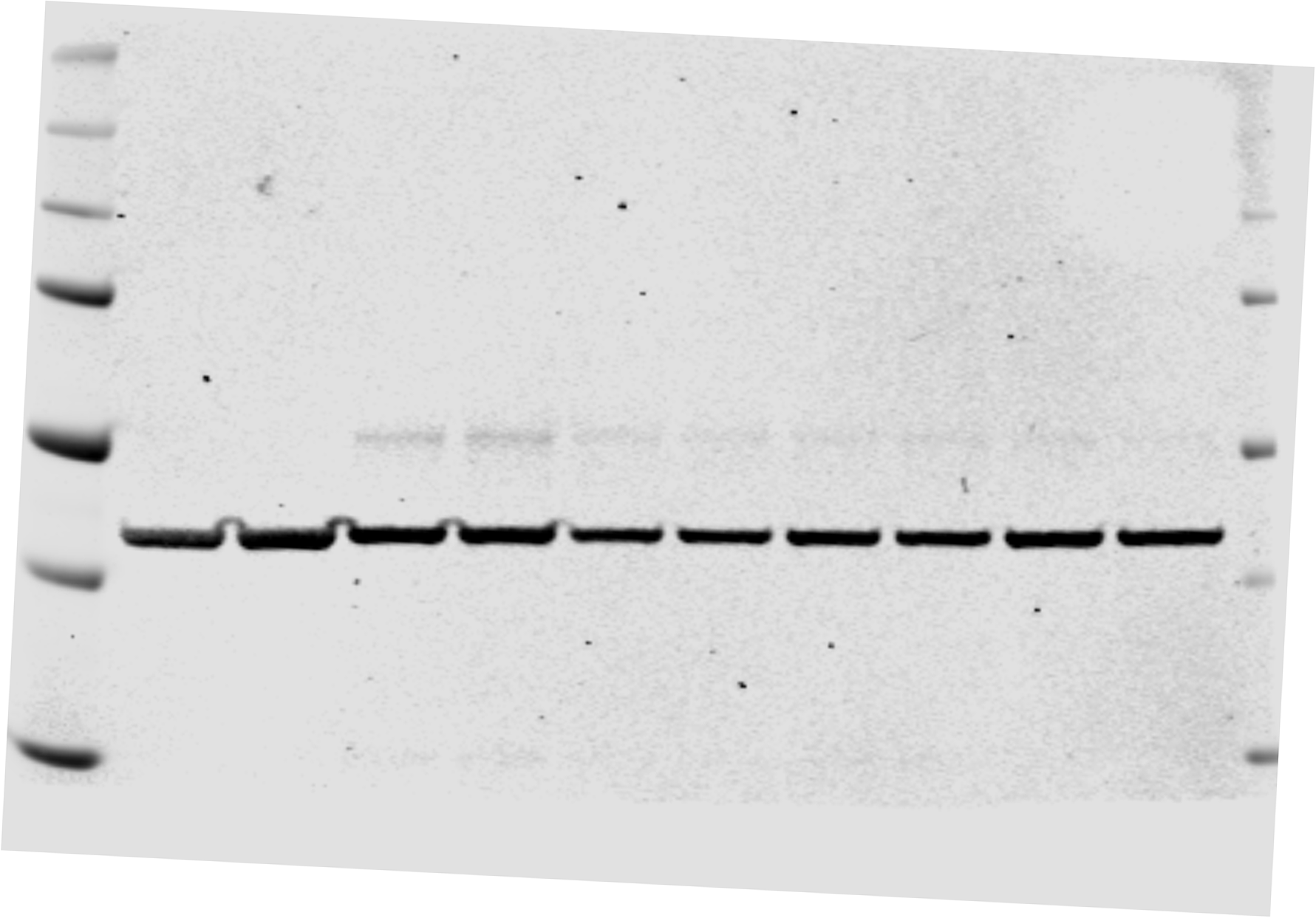

Supplement: Figure 4—source data 8. — The SCGs from the three mice of the same strain were pooled and processed by beads homogenization to acquire tissue lysates. 15 μg of the tissue lysate were used for actin β (42 kDa) immunoblotting analysis as described in the ‘Immunoblotting’ section. The right eight lanes of the raw image (lysate from SCG tissues) were used for Figure 4H to show actin β protein in the SCGs. The left two lanes of the raw immunoblotting image represent the lysates of soluble fraction (RIPA lysis buffer extracted) of whole-brain tissues from the ChatCre and chTKO mice, respectively. A detailed description of the raw images is shown in Source data 1. [file elife-73360-fig4-data8.zip › Tang_25-08-2021-RA-eLife-73360R1_Figure_4_source_data_8.png]

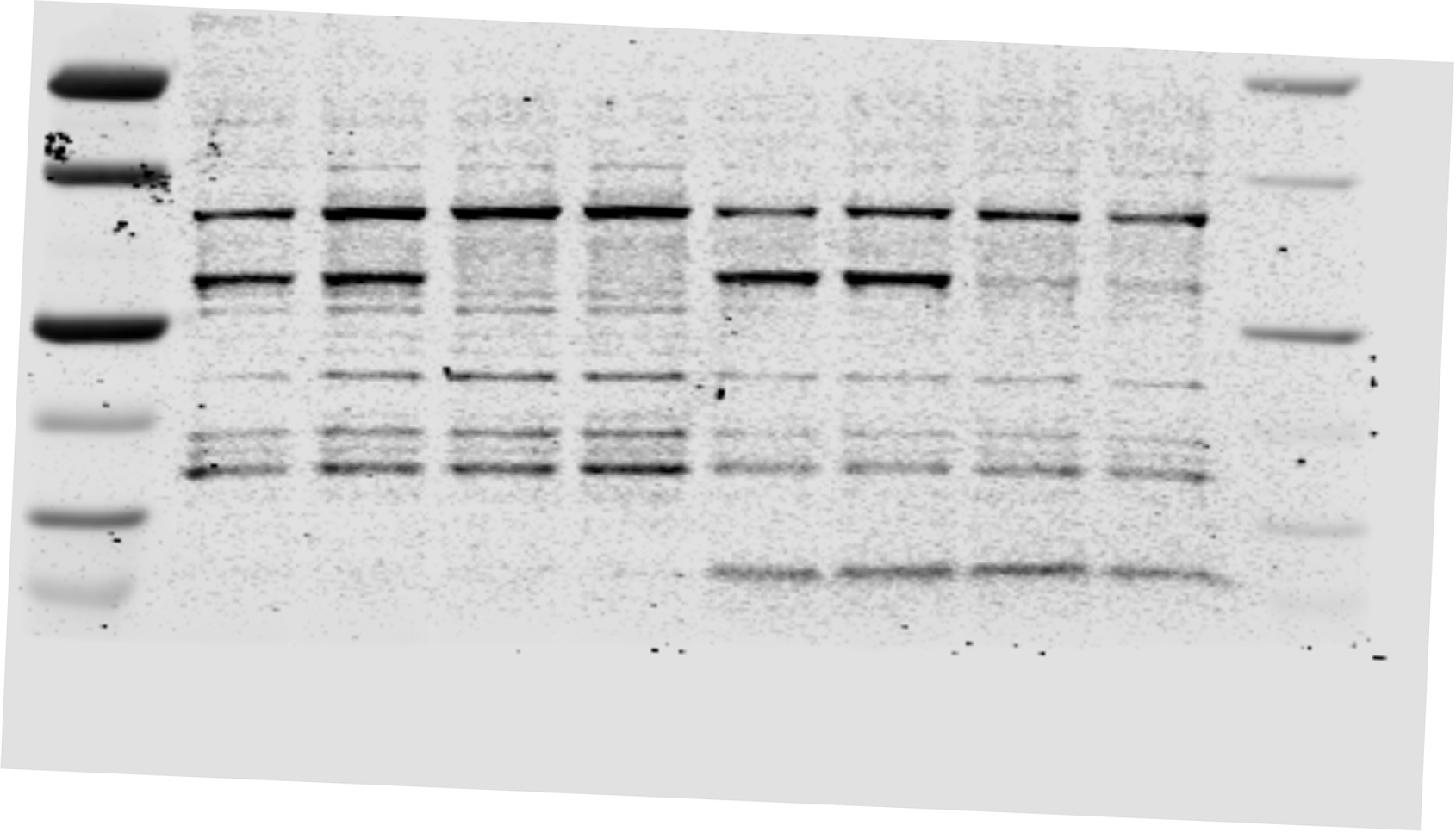

Supplement: Figure 6—source data 1. — The left four lanes of the raw image (lysate from neuroblastoma cells) were used for Figure 6E to confirm the efficiency of TIGAR protein loss in the SH-SY5Y neuroblastoma TKO cells. The right four lanes of the raw image represent the TIGAR immunoblotting of the cell lysates from 7-day differentiated neuroblastoma cells. A detailed description of the raw images is shown in Source data 1. [file elife-73360-fig6-data1.zip › Tang_25-08-2021-RA-eLife-73360R1_Figure_6_source_data_1.png]

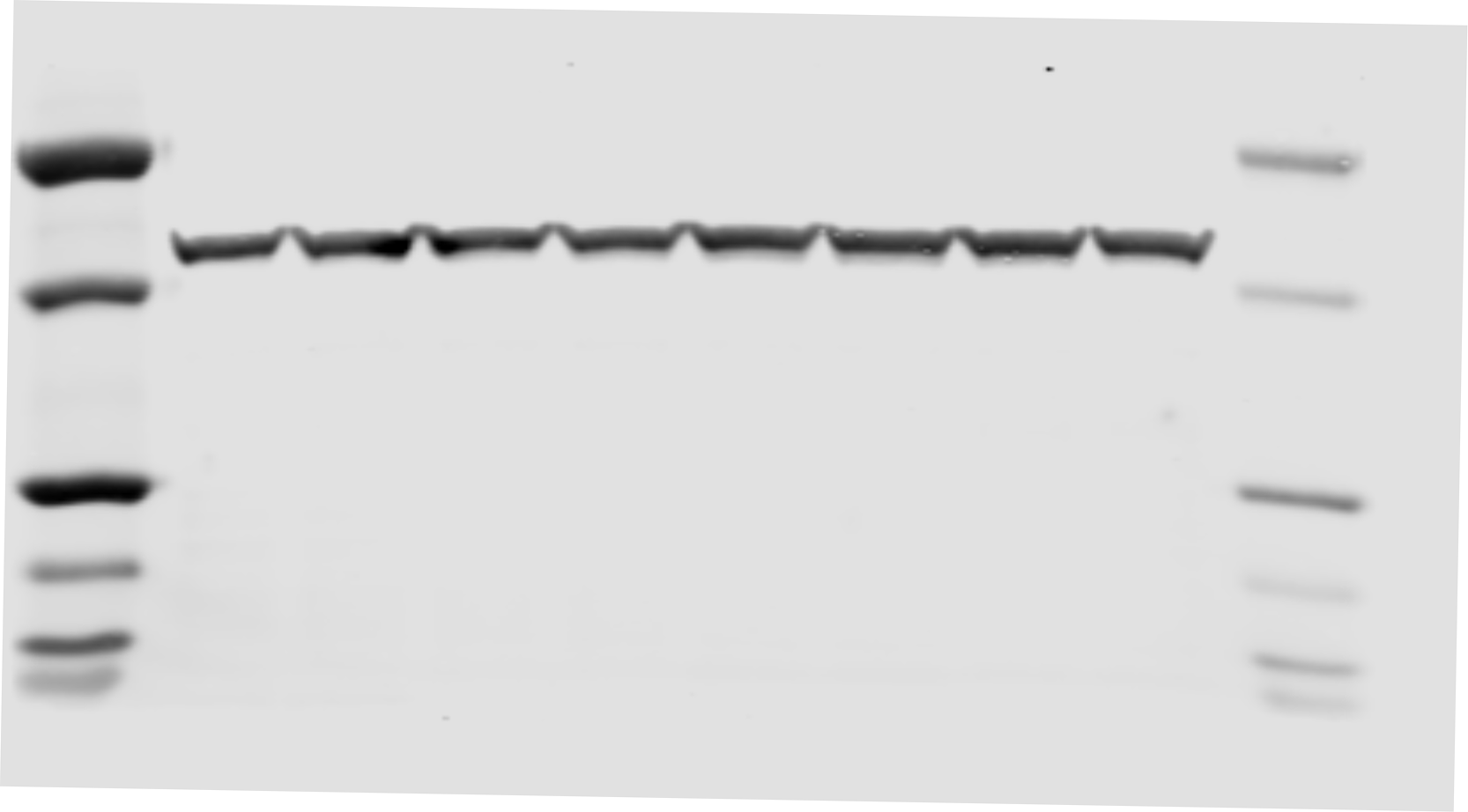

Supplement: Figure 6—source data 2. — The left four lanes of the raw image (lysate from neuroblastoma cells) were used for Figure 6E to show actin β protein in the SH-SY5Y neuroblastoma cells. The right four lanes of the raw image represent the actin β immunoblotting of the cell lysates from 7-day differentiated neuroblastoma cells. A detailed description of the raw images is shown in Source data 1. [file elife-73360-fig6-data2.zip › Tang_25-08-2021-RA-eLife-73360R1_Figure_6_source_data_2.png]

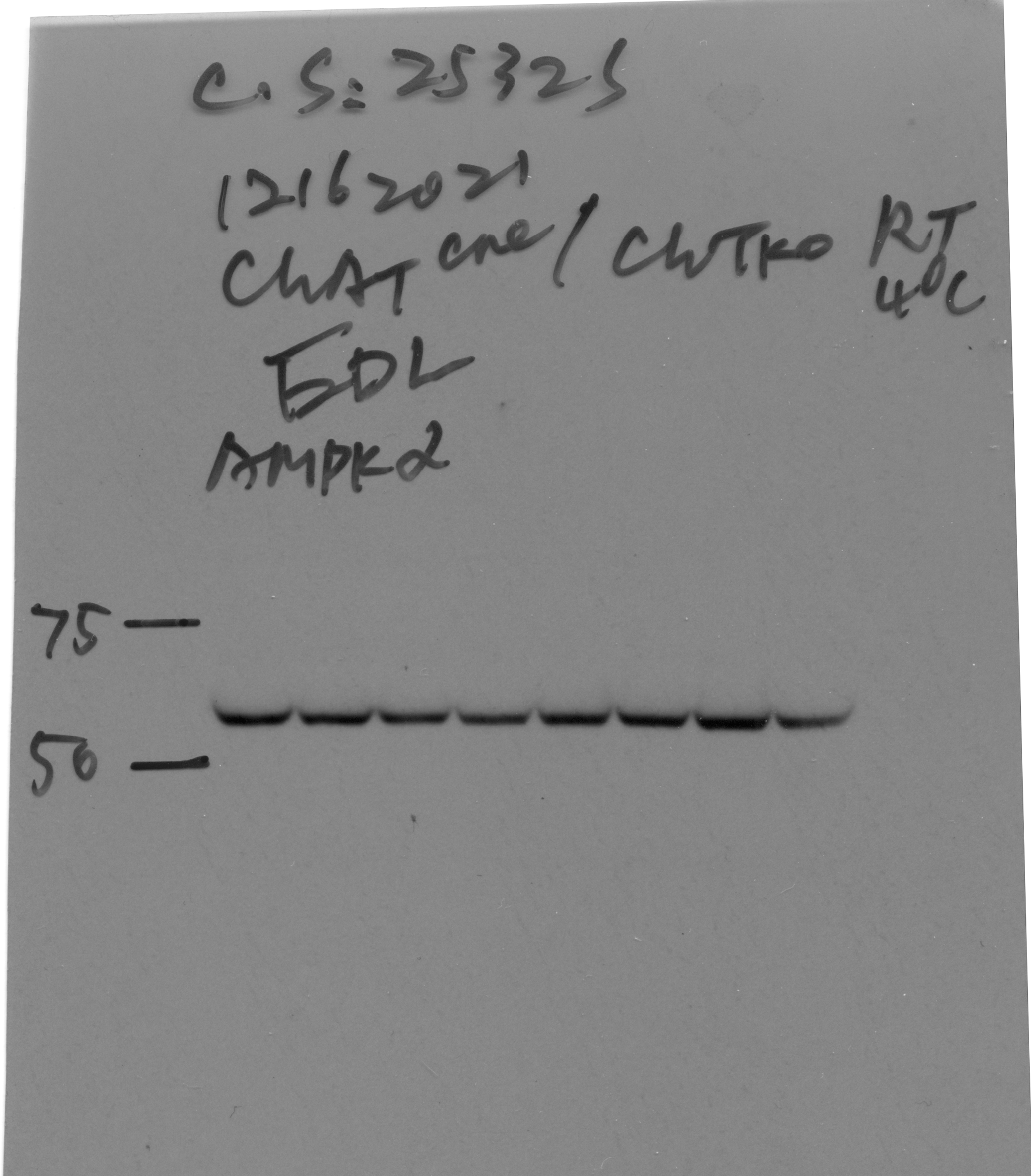

Supplement: Figure 7—source data 1. — The raw image was used for Figure 7F. A detailed description of the raw images is shown in Source data 1. [file elife-73360-fig7-data1.zip › Tang_25-08-2021-RA-eLife-73360R1_Figure_7_source_data_1.png]

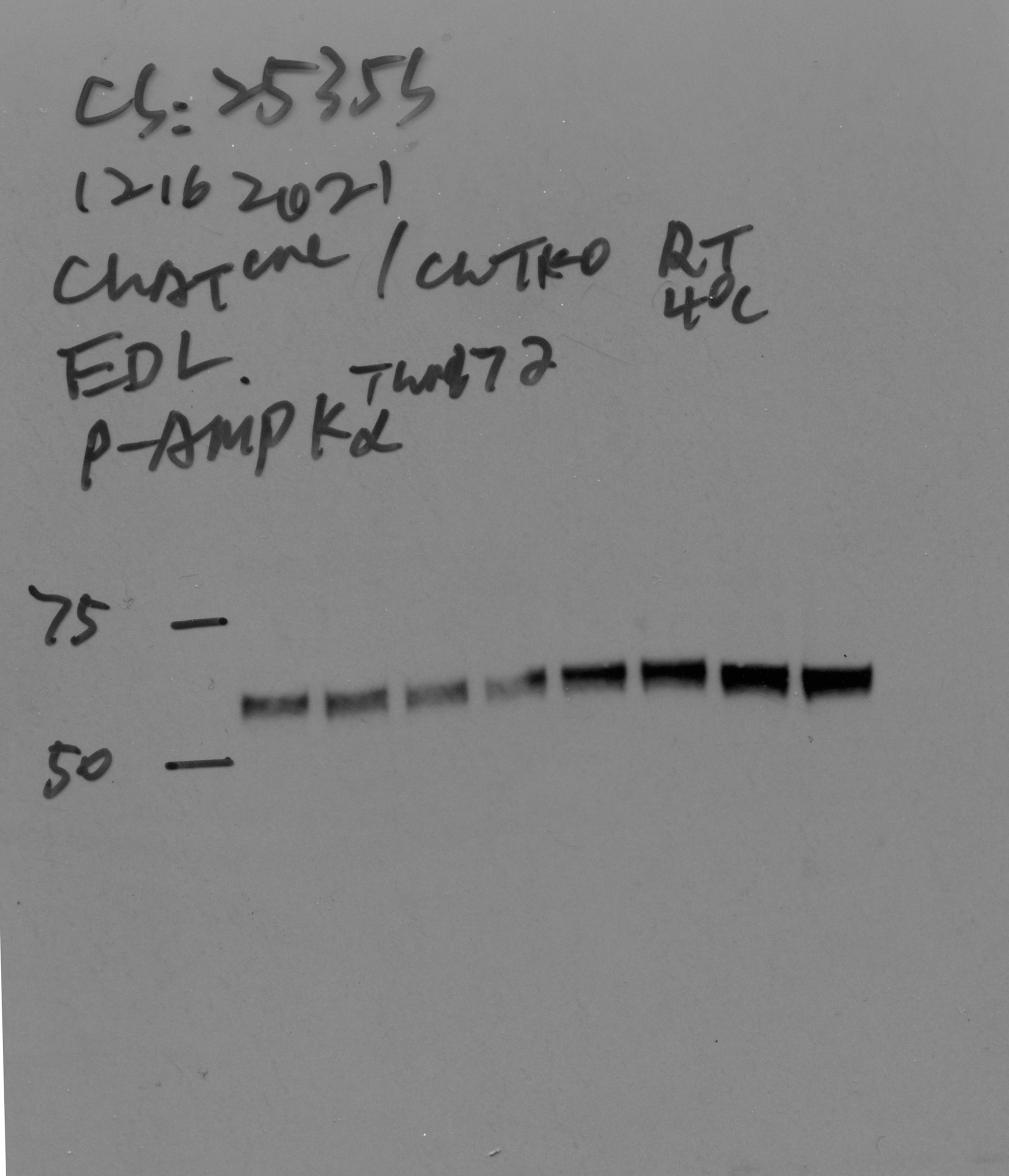

Supplement: Figure 7—source data 2. — The raw image was used for Figure 7F. A detailed description of the raw images is shown in Source data 1. [file elife-73360-fig7-data2.zip › Tang_25-08-2021-RA-eLife-73360R1_Figure_7_source_data_2.png]
