# Supplementary material for: TIGAR deficiency enhances skeletal muscle thermogenesis by increasing neuromuscular junction cholinergic signaling
Source: eLife. 2022 Mar 7;11:e73360. doi: 10.7554/eLife.73360 (PMC8947760; doi:10.7554/eLife.73360)
Supplement: Source data 1. [file elife-73360-data1.pdf]

Figure 1-source data 1

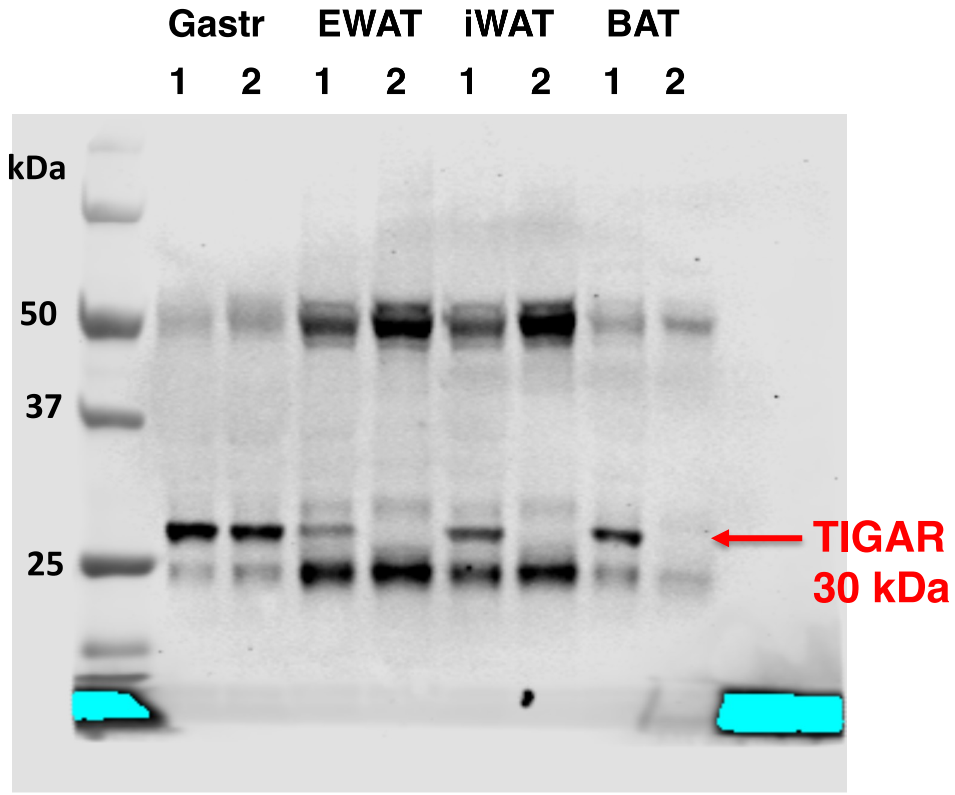

Gastr: gastrocnemius muscle
EWAT: epididymal adipose tissue
iWAT: inguinal adipose tissue
BAT: interscapular brown adipose tissue

1, Tigar$^{fl/fl}$
2, Tigar$^{fl/fl}$/Adipoq^{Cre}

Figure 1-source data 1

Fresh gastrocnemius muscle (Gastr), epididymal adipose tissue (EWAT), inguinal white adipose tissue, and interscapular brown adipose tissue from *Tigar$^{fl/fl}$* and *Tigar$^{fl/}$fl/Adipoq^{Cre}* mice were collected and 30 μg of the tissue lysate were used for TIGAR (30 kDa) immunoblotting analysis as described in the Immunoblotting, Method details. The raw image was used for Figure 1C.

Figure 1-source data 2

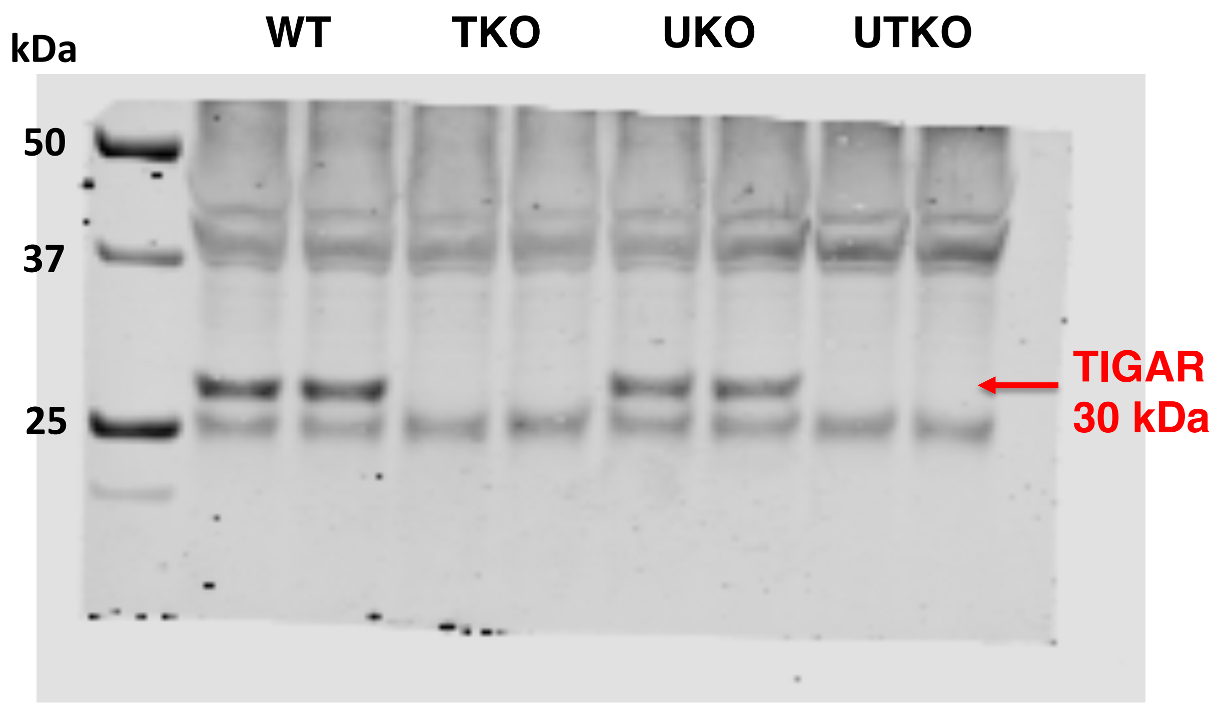

Figure 1-source data 2
Interscapular brown adipose tissues were collected from WT, TKO, UKO, and UTKO mice and 30 μg of the tissue lysate were used for TIGAR (30 kDa) immunoblotting analysis as described in the Immunoblotting, Method details. The raw image was used for Figure 1E.

Figure 1-source data 3

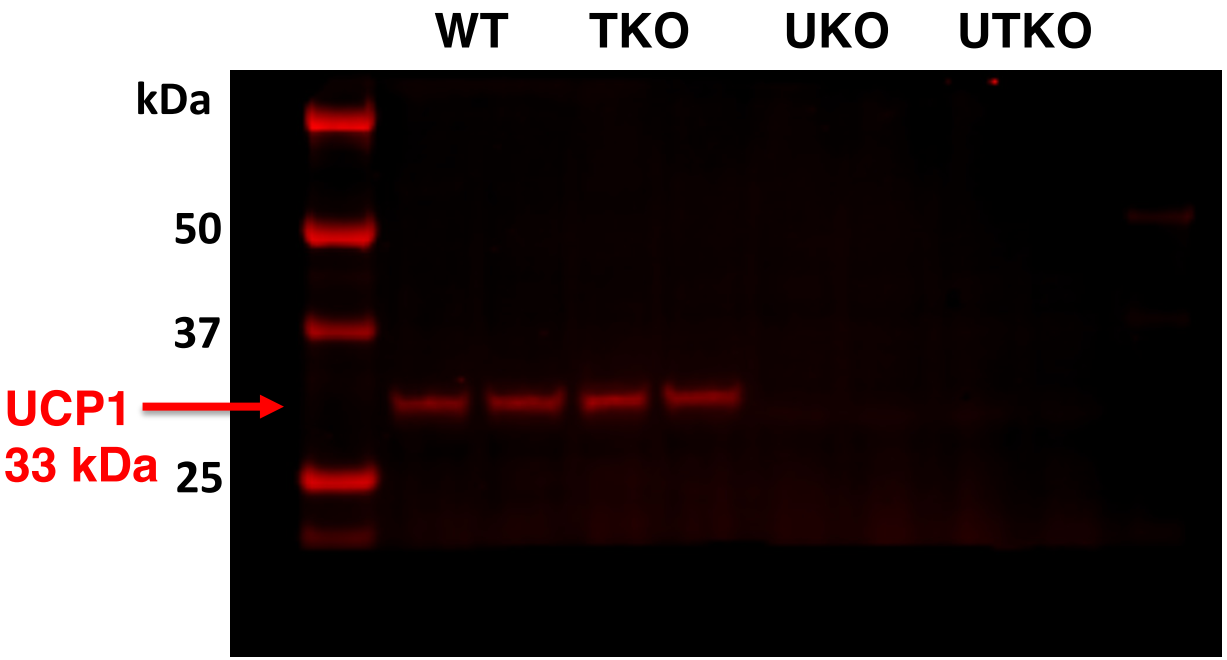

Figure 1-source data 3
Interscapular brown adipose tissues were collected from WT, TKO, UKO, and UTKO mice and 30 μg of the tissue lysate were used for UCP1 (33 kDa) immunoblotting analysis as described in the Immunoblotting, Method details. The raw image was used for Figure 1E. The raw image was used for Figure 1E.

Figure 1-source data 4

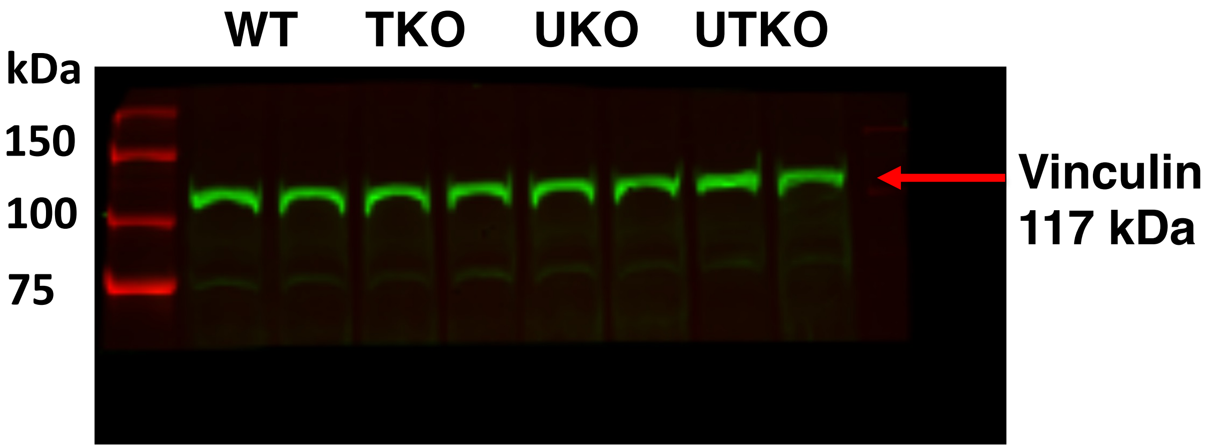

Figure 1-source data 4
Interscapular brown adipose tissues were collected from WT, TKO, UKO, and UTKO mice and 30 μg of the tissue lysate were used for vinculin (117 kDa) immunoblotting analysis as described in the Immunoblotting, Method details. The raw image was used for Figure 1E.

Figure 1-source data 5

Gastr: gastrocnemius muscle
EDL: Extensor digitorum longus muscle

1, Myl1^{Cre}
2, Myl1Cre/Tigar$^{fl/fl}$

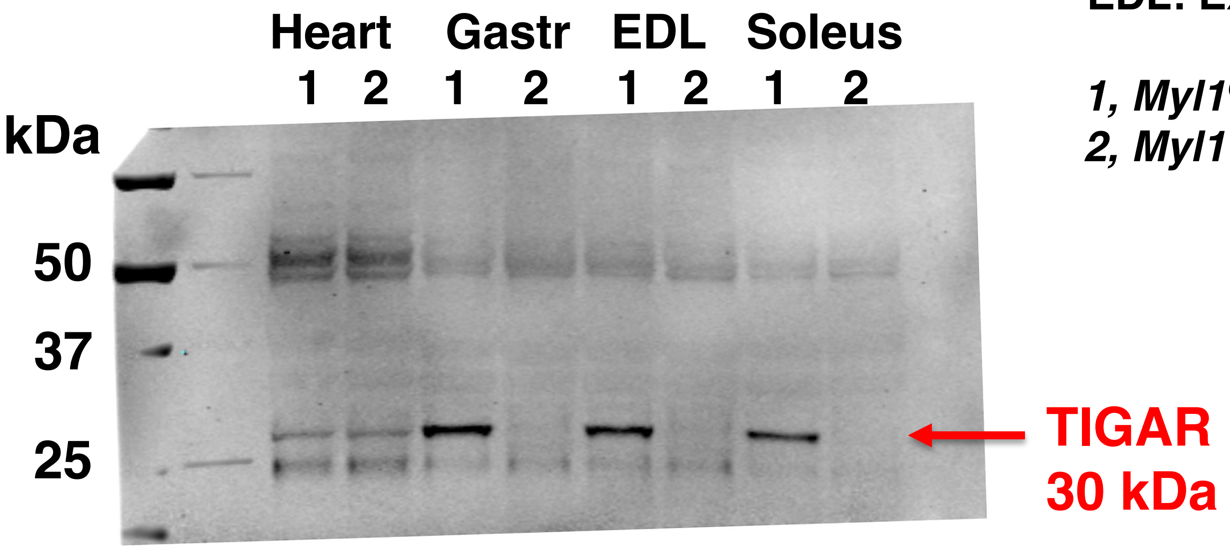

Figure 1-source data 5
Fresh heart, gastrocnemius muscle (Gastr), extensor digitorum longus (EDL muscle), and soleus muscle were collected from *Myl1^{Cre}* and *Myl1^{Cre}/Tigar$^{fl/fl}$* mice and 30 μg of the tissue lysate were used for TIGAR (30 kDa) immunoblotting analysis as described in the Immunoblotting, Method details. The raw image was used for Figure 1 G.

Figure 1-source data 6

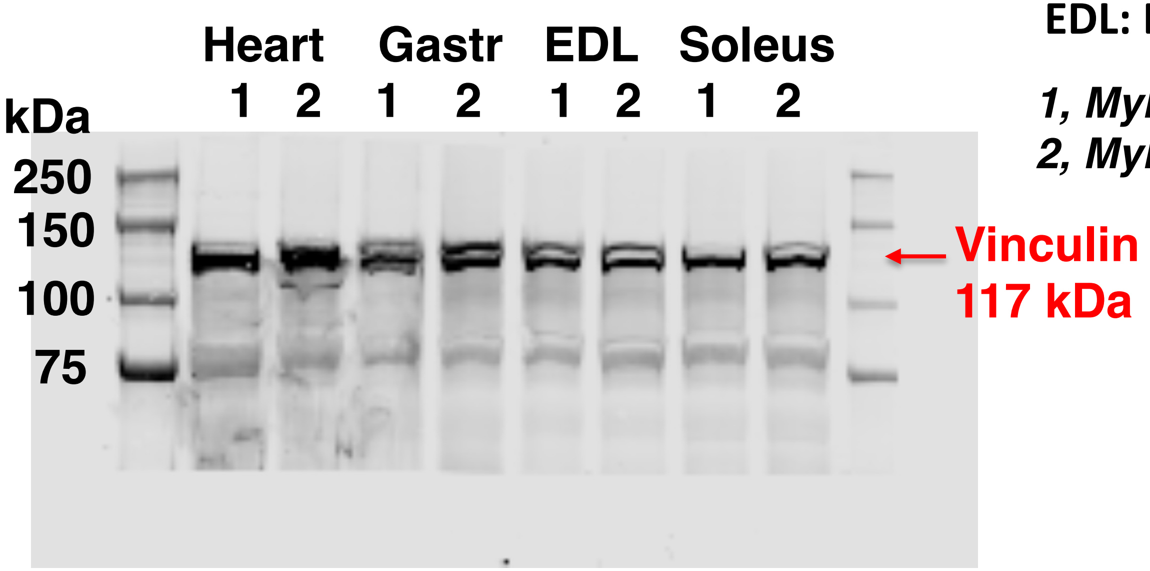

Gastr: gastrocnemius muscle
EDL: Extensor digitorum longus muscle

1, Myl1^{Cre}
2, Myl1Cre/Tigar$^{fl/fl}$

Figure 1-source data 6
Fresh heart, gastrocnemius muscle (Gastr), extensor digitorum longus (EDL muscle), and soleus muscle were collected from *Myl1*^{Cre} and *Myl1*^{Cre}*/Tigar*$^{fl/fl}$ mice and 30 μg of the tissue lysate were used for vinculin (117 kDa) immunoblotting analysis as described in the Immunoblotting, Method details. The raw image was used for Figure 1

Figure 4-source data 1

Ambient T.: ambient temperature (21-23°C)

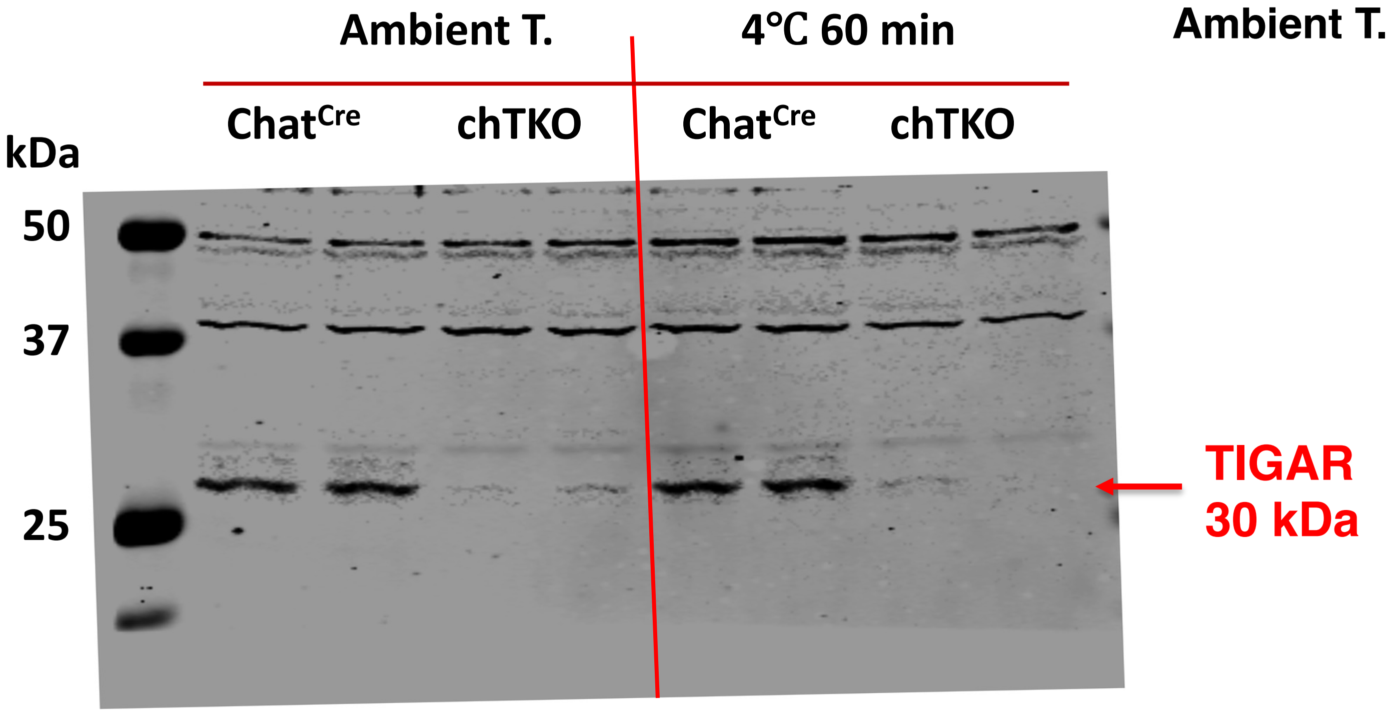

Figure 4-source data 1

The superior cervical ganglion (SCG) tissues were collected and snap frozen in liquid nitrogen from *Chat*^{Cre} and chTKO mice, as shown in the Figure 4-figure supplement for SCG dissection. The SCGs from three mice of the same strain were pooled and processed by beads homogenization to acquire tissue lysates. 15 µg of the tissue lysate were used for TIGAR (30 kDa) immunoblotting analysis as described in the Immunoblotting, Method details. The left four lanes of the raw image (lysate from the mice at ambient temperature) were used for Figure 4 B to confirm the efficiency of TIGAR protein loss in SCG of the chTKO mice.

Figure 4-source data 2

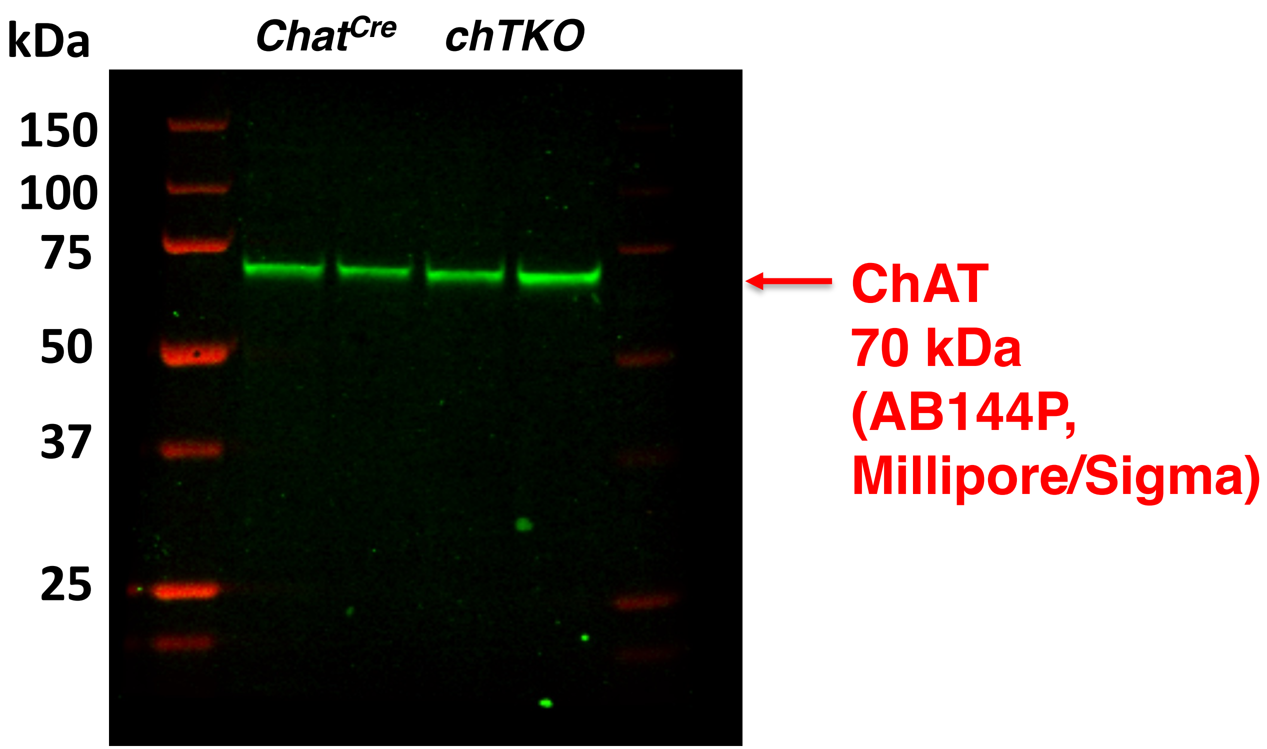

Figure 4-source data 2
The superior cervical ganglion (SCG) tissues were collected and snap frozen in liquid nitrogen from *Chat*^{Cre} and chTKO mice, as shown in the Figure 4-figure supplement for SCG dissection. The SCGs from three mice of the same strain were pooled and processed by beads homogenization to acquire tissue lysates. 15 μg of the tissue lysate were used for choline acetyltransferase (ChAT, 70 kDa) immunoblotting analysis as described in the Immunoblotting, Method details. The left four lanes of the raw image (lysate from the mice at ambient temperature) were used for Figure 4 B.

Figure 4-source data 3

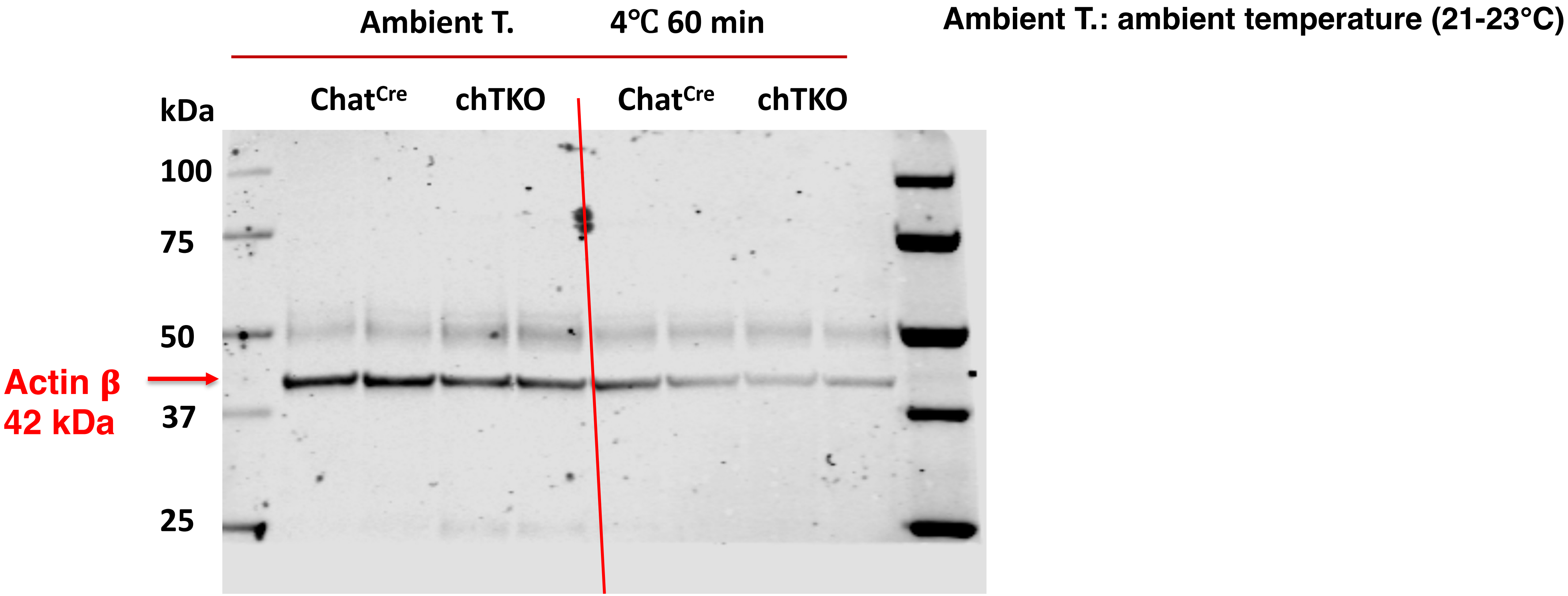

Figure 4-source data 3
The superior cervical ganglion (SCG) tissues were collected and snap frozen in liquid nitrogen from *Chat*^{Cre} and chTKO mice, as shown in the Figure 4-figure supplement for SCG dissection. The SCGs from three mice of the same strain were pooled and processed by beads homogenization to acquire tissue lysates. 15 μg of the tissue lysate were used for Actin **β** (42 kDa) immunoblotting analysis as described in the Immunoblotting, Method details. The left four lanes of the raw image (lysate from the mice at ambient temperature) were used for Figure 4 B.

Figure 4-source data 4

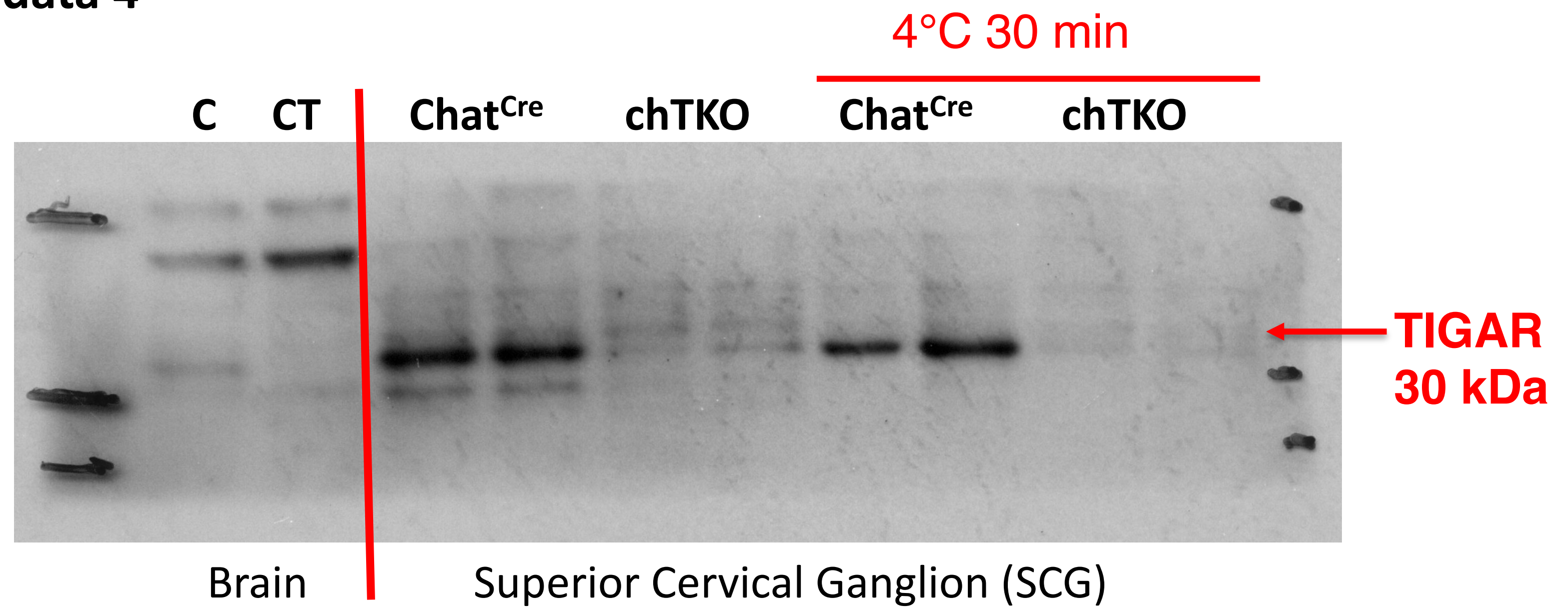

Figure 4-source data 4

The superior cervical ganglion (SCG) tissues were collected and snap frozen in liquid nitrogen from both ambient temperature housed and 4°C 30 minutes exposed *Chat*^{Cre} and chTKO mice, as shown in the Figure 4-figure supplement for SCG dissection. The SCGs from three mice of the same strain were pooled and processed by beads homogenization to acquire tissue lysates. 15 μg of the tissue lysate were used for TIGAR (30 kDa) immunoblotting analysis as described in the Immunoblotting, Method details. The right eight lanes of the raw image (lysate from SCG tissues) was used for Figure 4 H to confirm the efficiency of TIGAR protein loss in SCG of the chTKO mice. The left two lanes of the raw immunoblotting image represent the lysates of soluble fraction (RIPA lysis buffer extracted) of whole brain tissues from the *Chat*^{Cre} and chTKO mice, respectively.

Figure 4-source data 5

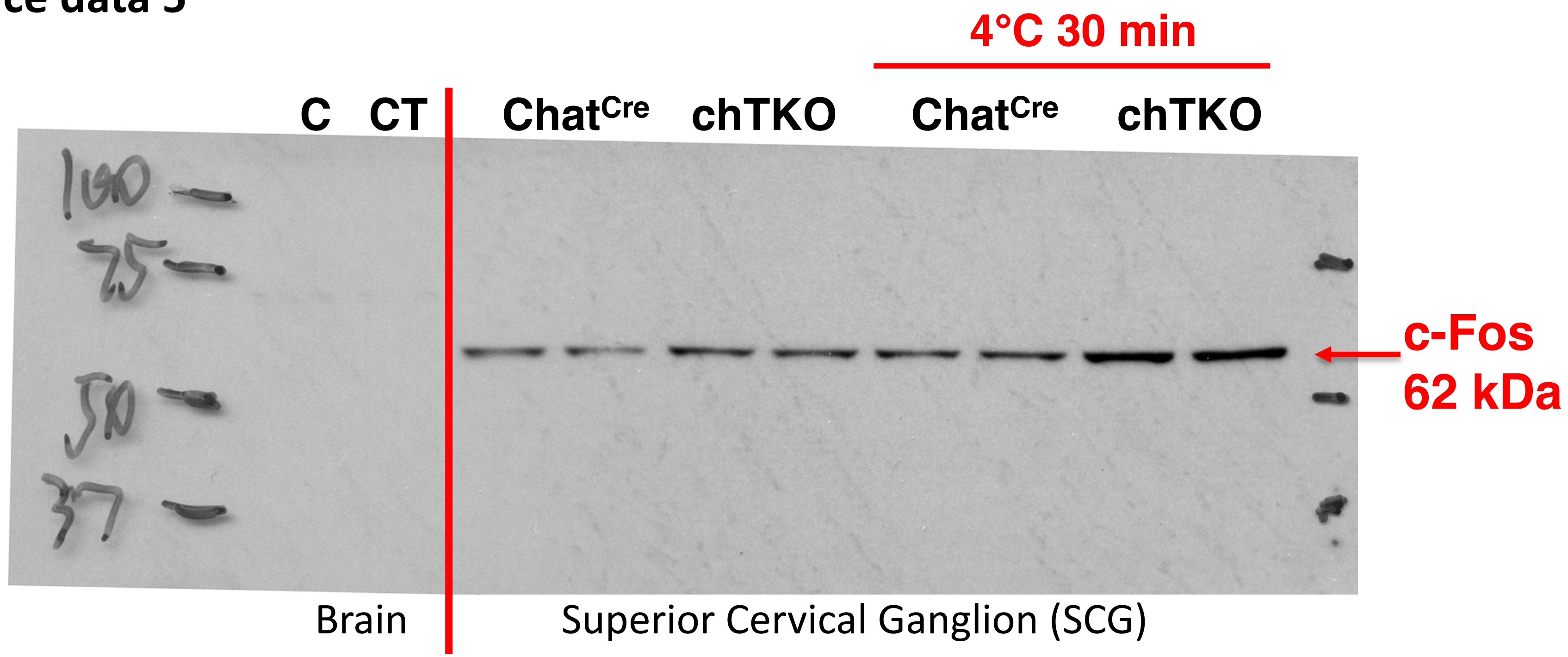

Figure 4-source data 5

The superior cervical ganglion (SCG) tissues were collected and snap frozen in liquid nitrogen from both ambient temperature housed and 4°C 30 minutes exposed *Chat*^{Cre} and chTKO mice, as shown in the Figure 4-figure supplement for SCG dissection. The SCGs from three mice of the same strain were pooled and processed by beads homogenization to acquire tissue lysates. 15 μg of the tissue lysate were used for c-Fos (62 kDa) immunoblotting analysis as described in the Immunoblotting, Method details. The right eight lanes of the raw image (lysate from SCG tissues) was used for Figure 4 H to show the increase of c-Fos protein in SCG of chTKO mice under cold exposed. The left two lanes of the raw immunoblotting image represent the lysates of soluble fraction (RIPA lysis buffer extracted) of whole brain tissues from *Chat*^{Cre} and chTKO mice, respectively.

Figure 4-source data 6

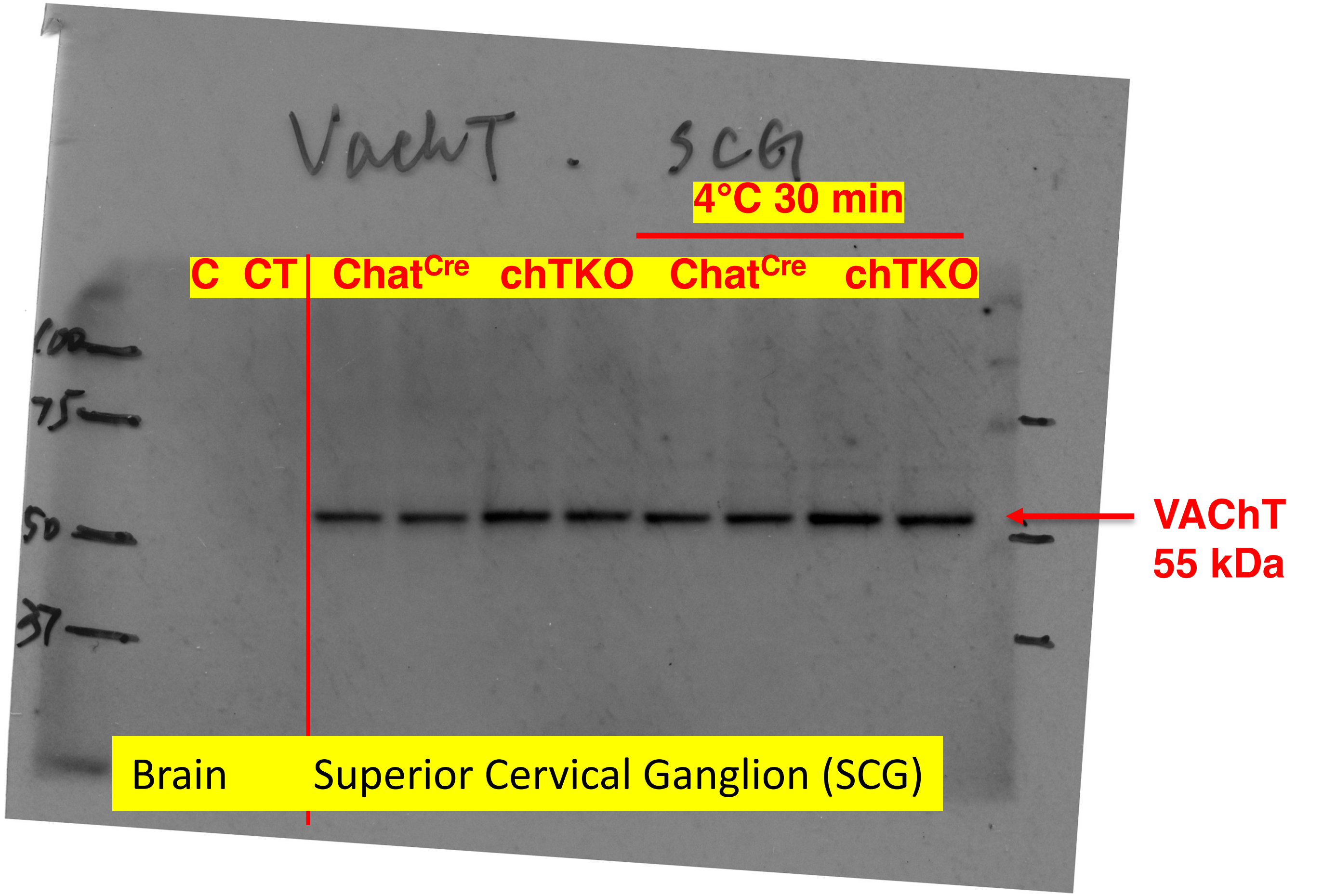

Figure 4-source data 6

The superior cervical ganglion (SCG) tissues were collected and snap frozen in liquid nitrogen from both ambient temperature housed and 4°C 30 minutes exposed *Chat*^{Cre} and chTKO mice, as shown in the Figure 4-figure supplement for SCG dissection. The SCGs from three mice of the same strain were pooled and processed by beads homogenization to acquire tissue lysates. 15 μg of the tissue lysate were used for vesicular acetylcholine transporter (VAChT, 55 kDa) immunoblotting analysis as described in the Immunoblotting, Method details. The right eight lanes of the raw image (lysate from SCG tissues) were used for Figure 4 H to show VAChT protein in the SCGs. The left two lanes of the raw immunoblotting image represent the lysates of soluble fraction (RIPA lysis buffer extracted) of whole brain tissues from *Chat*^{Cre} and chTKO mice, respectively.

Figure 4-source data 7

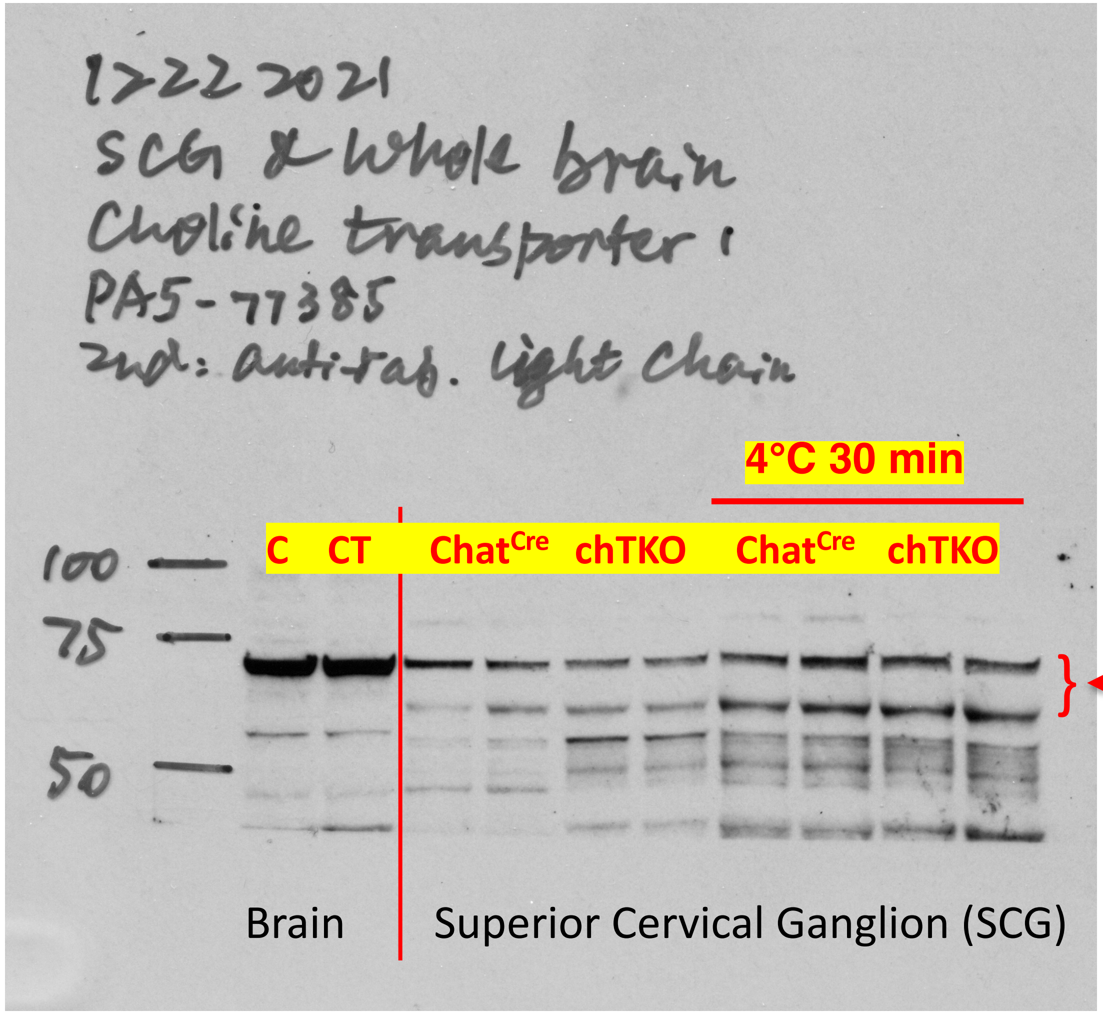

Figure 4-source data 7

The superior cervical ganglion (SCG) tissues were collected and snap frozen in liquid nitrogen from both ambient temperature housed and 4°C 30 minutes exposed *Chat*^{Cre} and chTKO mice, as shown in the Figure 4-figure supplement for SCG dissection. The SCGs from three mice of the same strain were pooled and processed by beads homogenization to acquire tissue lysates. 15 μg of the tissue lysate were used for choline transporter (ChT, 55 kDa) immunoblotting analysis as described in the Immunoblotting, Method details. The right eight lanes of the raw image (lysate from SCG tissues) were used for Figure 4 H to show ChT protein in that 63 and 70 kDa bands were observed in the SCGs. The left two lanes of the raw immunoblotting image represent the lysates of soluble fraction (RIPA lysis buffer extracted) of whole brain tissues from *Chat*^{Cre} and chTKO mice, respectively.

Choline transporter (ChT)
(SLC5A7, extracellular)
63-70 kDa observed

Cat# PA5-77385,
ThermoFisher
Scientific

Figure 4-source data 8

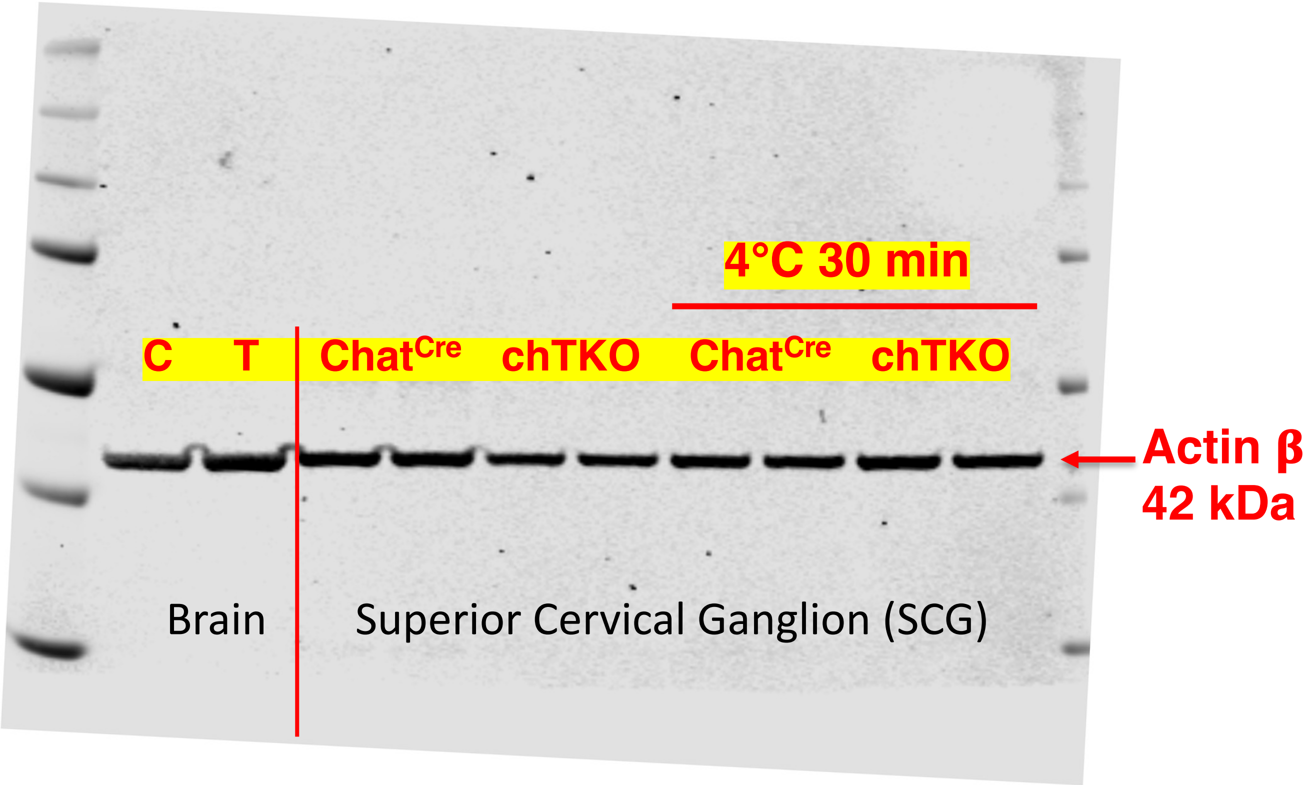

Figure 4-source data 8

The superior cervical ganglion (SCG) tissues were collected and snap frozen in liquid nitrogen from both ambient temperature housed and 4°C 30 minutes exposed *Chat*^{Cre} and chTKO mice, as shown in the Figure 4-figure supplement for SCG dissection. The SCGs from three mice of the same strain were pooled and processed by beads homogenization to acquire tissue lysates. 15 μg of the tissue lysate were used for actin β (42 kDa) immunoblotting analysis as described in the Immunoblotting, Method details. The right eight lanes of the raw image (lysate from SCG tissues) were used for Figure 4 H to show actin β protein in the SCGs. The left two lanes of the raw immunoblotting image represent the lysates of soluble fraction (RIPA lysis buffer extracted) of whole brain tissues from *Chat*^{Cre} and chTKO mice, respectively.

Figure 6-source data 1

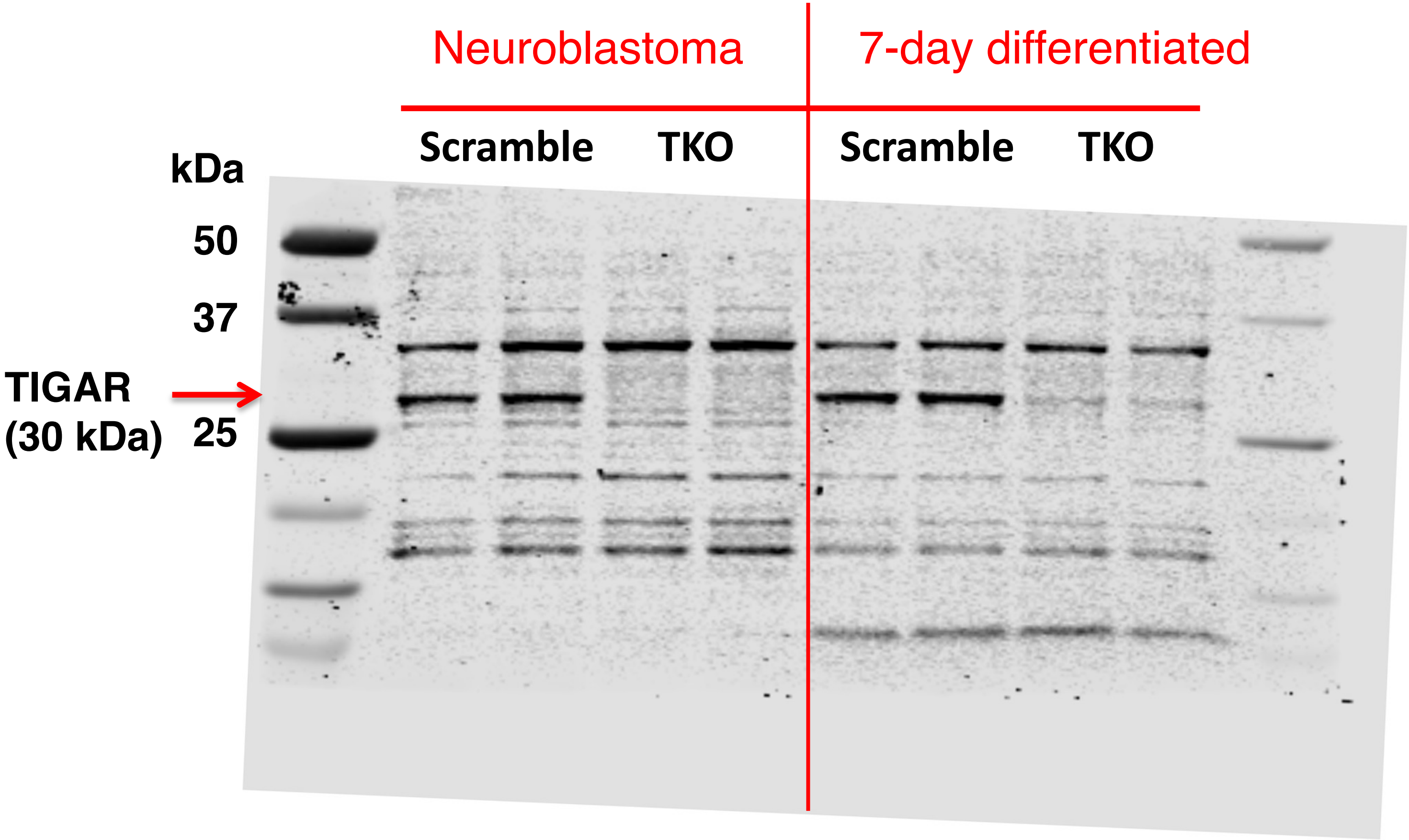

Figure 6-source data 1
The culture SH-SY5Y cells were collected from both scrambled and Tigar knockout (TKO) cells and 30 μg of the cell lysates were used for TIGAR (30 kDa) immunoblotting analysis as described in the Immunoblotting, Method details. The left four lanes of the raw image (lysate from neuroblastoma cells) were used for Figure 6 E to confirm the efficiency of TIGAR protein loss in the SH-SY5Y neuroblastoma TKO cells. The right four lanes of the raw image represent the TIGAR immunoblotting of the cell lysates from seven day differentiated neuroblastoma cells.

Figure 6-source data 2

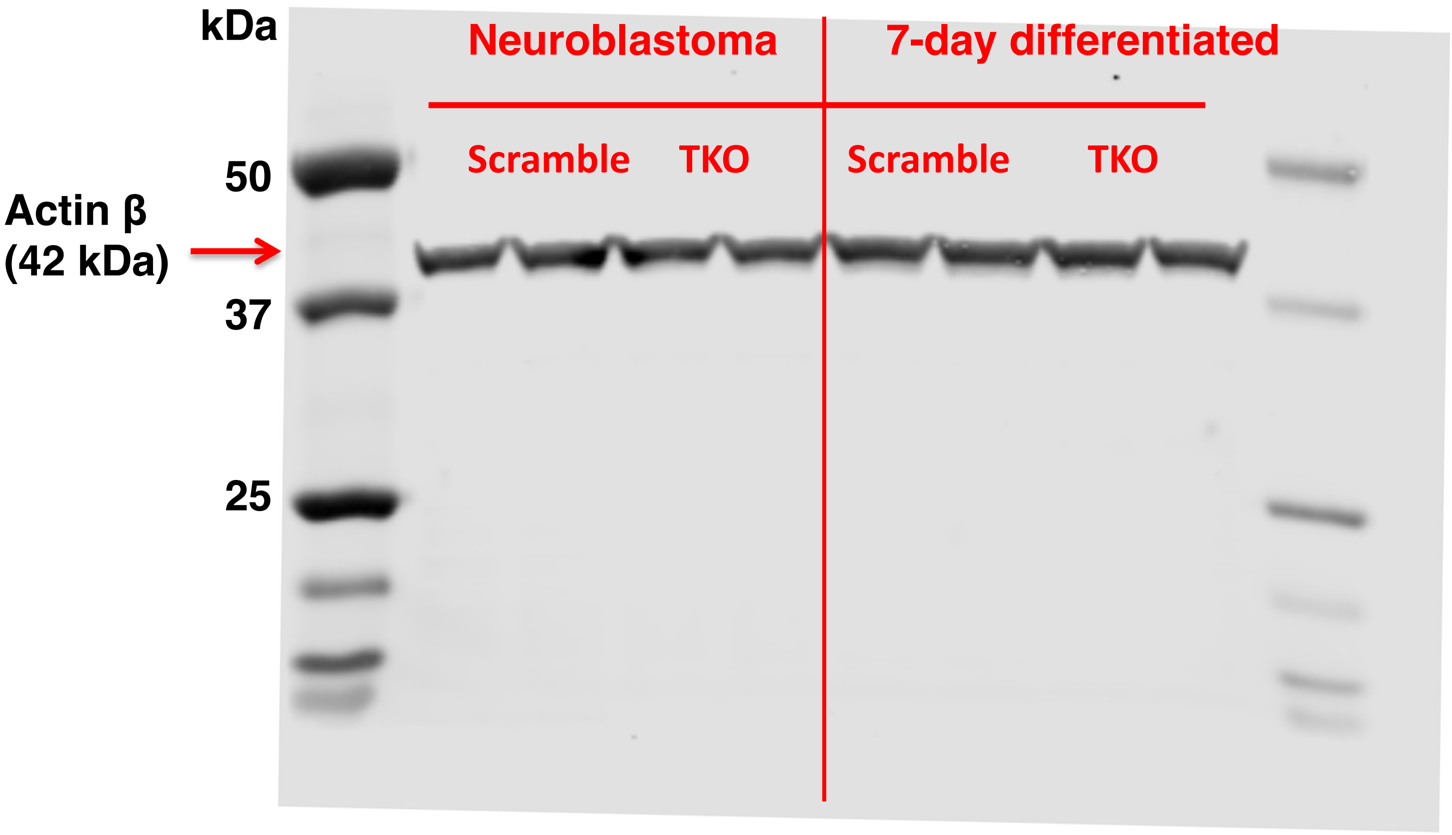

Figure 6-source data 2
The culture SH-SY5Y cells were collected from both scrambled and Tigar knockout (TKO) cells and 30 μg of the cell lysates were used for actin β (42 kDa) immunoblotting analysis as described in the Immunoblotting, Method details. The left four lanes of the raw image (lysate from neuroblastoma cells) were used for Figure 6 E to show actin β protein in the in the SH-SY5Y neuroblastoma cells. The right four lanes of the raw image represent the actin β immunoblotting of the cell lysates from seven day differentiated neuroblastoma cells.

Figure 7-source data 1

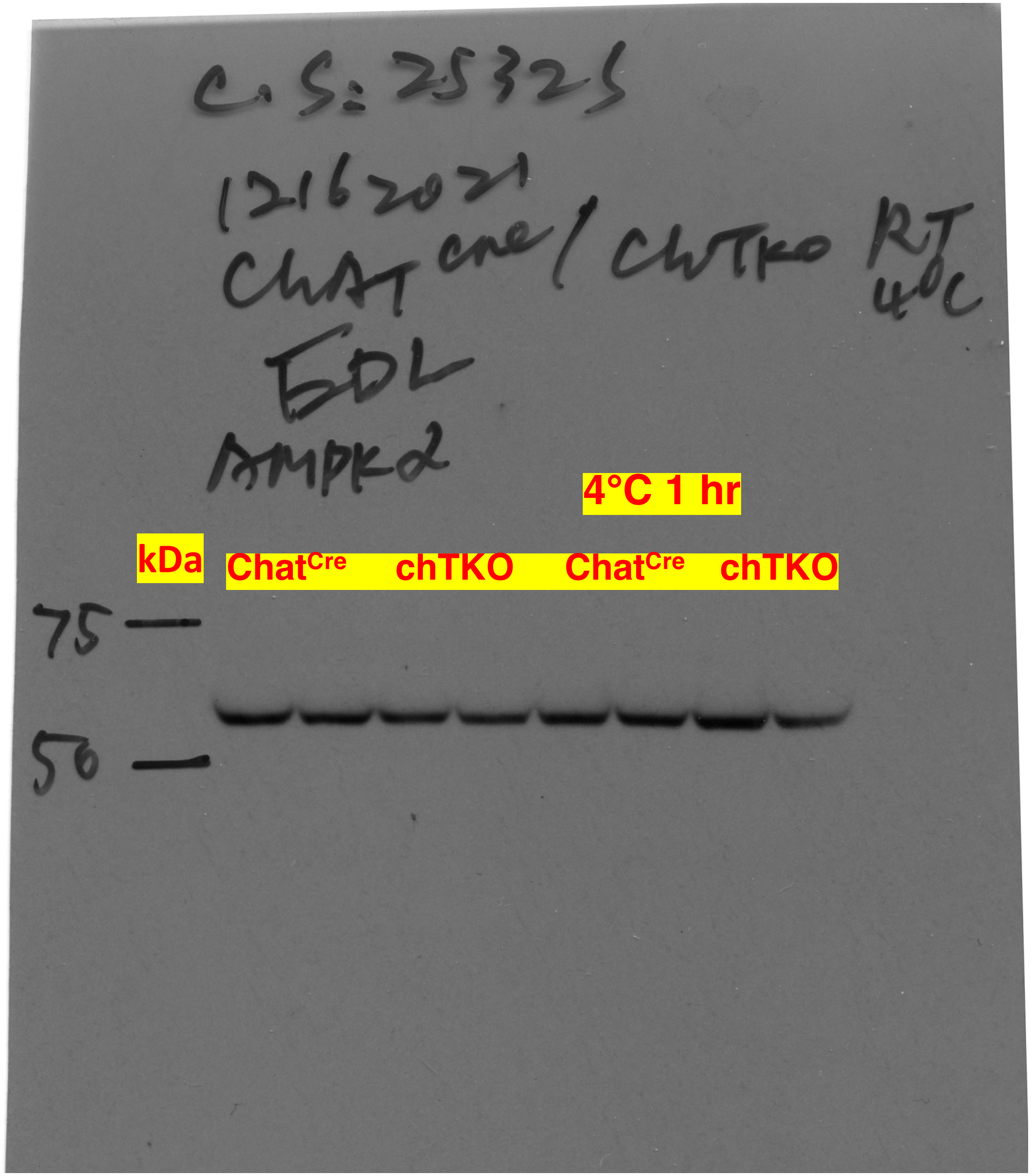

Figure 7-source data 1
The extensor digitorum longus (EDL) muscles were collected from both ambient temperature housed and 4°C 1 hour exposed *Chat*^{Cre} and chTKO mice and 30 μg of the tissue lysate were used for AMPKα (62 kDa) immunoblotting analysis as described in the Immunoblotting, Method details. The raw image was used for Figure 7F.

Figure 7-source data 2

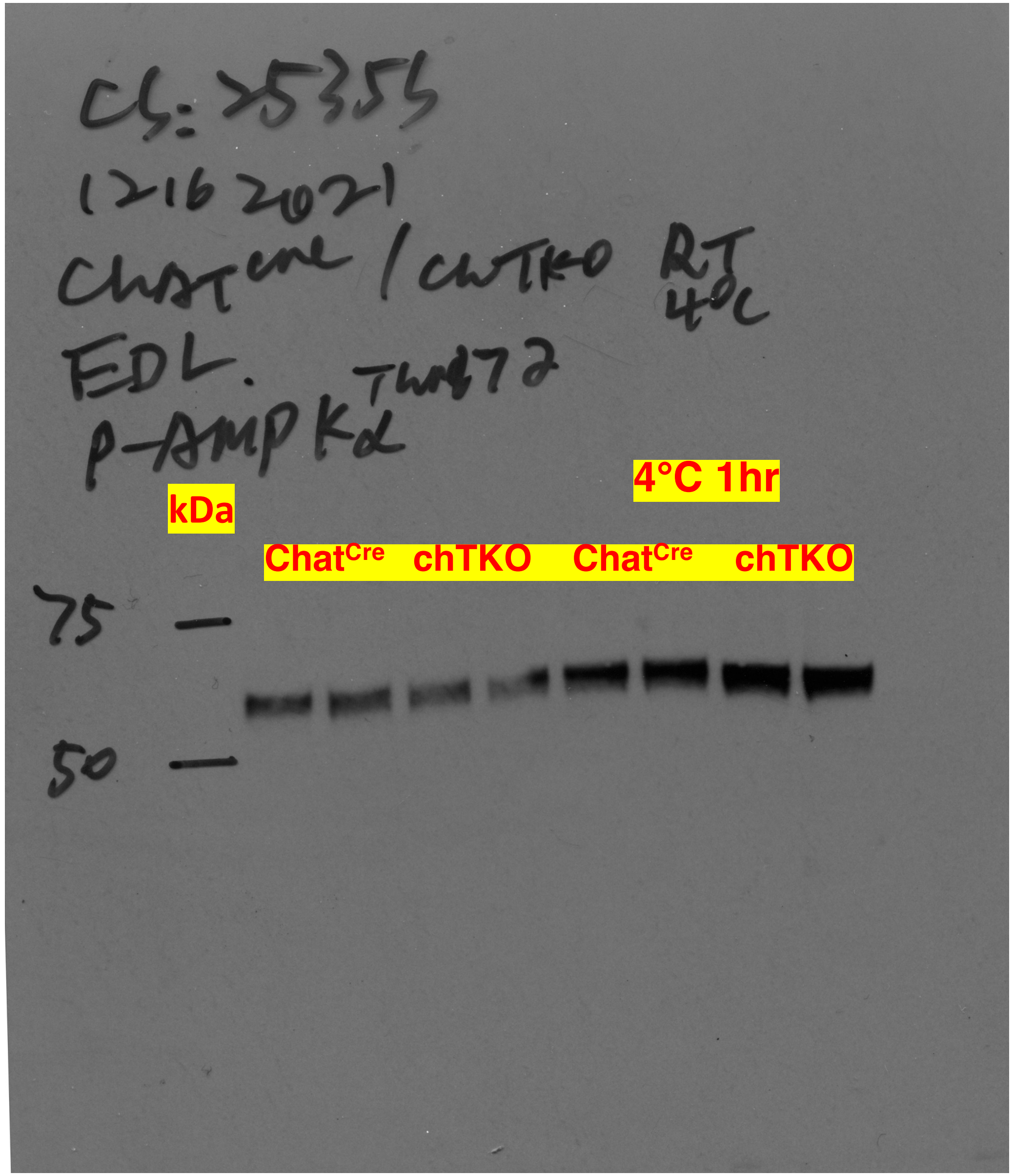

Figure 7-source data 2
The extensor digitorum longus (EDL) muscles were collected from both ambient temperature housed and 4°C 1 hour exposed *Chat*^{Cre} and chTKO mice and 30 μg of the tissue lysate were used for phospho-^{Thr172}AMPKα (62 kDa) immunoblotting analysis as described in the Immunoblotting, Method details. The raw image was used for Figure 7F.

Figure 1-figure supplement 1-source data 1

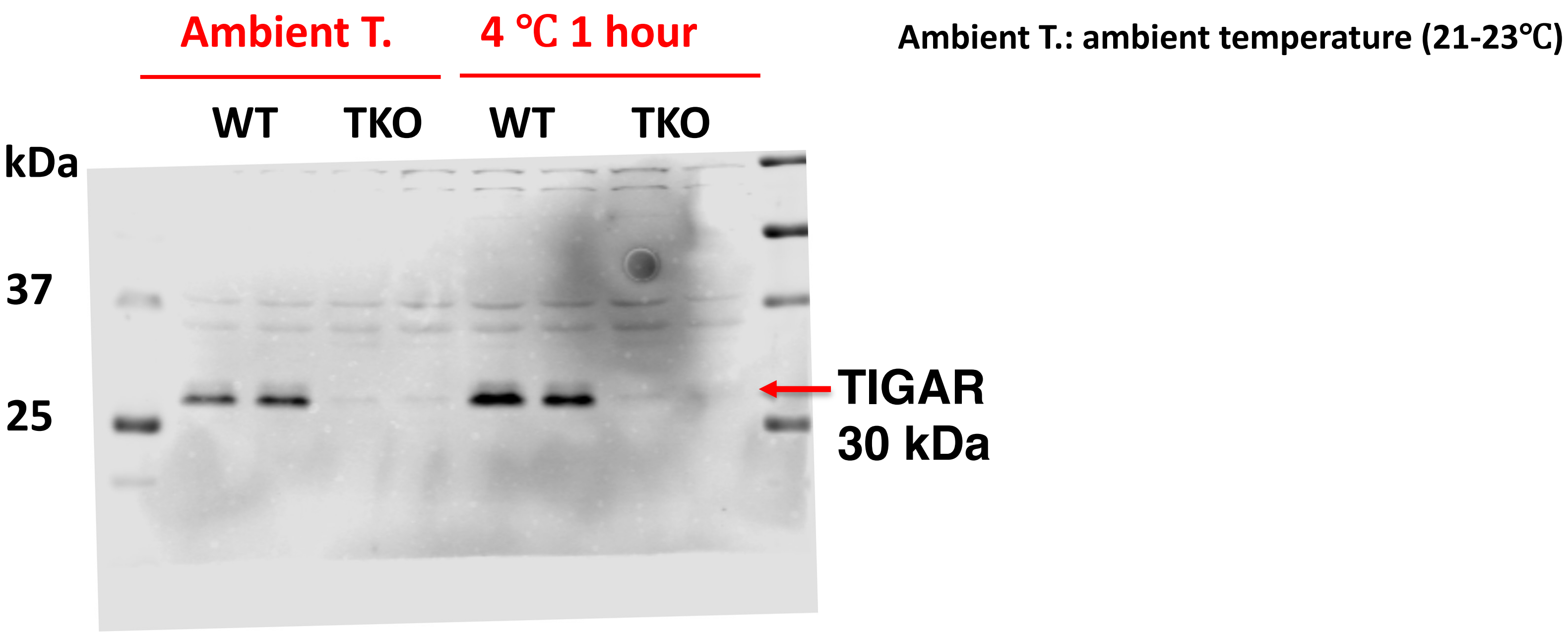

Figure 1-figure supplement 1-source data 1

Interscapular brown adipose tissues were collected from both ambient temperature housed and 4°C 1 hour exposed WT and TKO mice and 30 μg of the tissue lysate were used for TIGAR (30 kDa) immunoblotting analysis as described in the Immunoblotting, Method details. The raw image was used for Figure 1-figure supplement 1 B.

Figure 1-figure supplement 1-source data 2

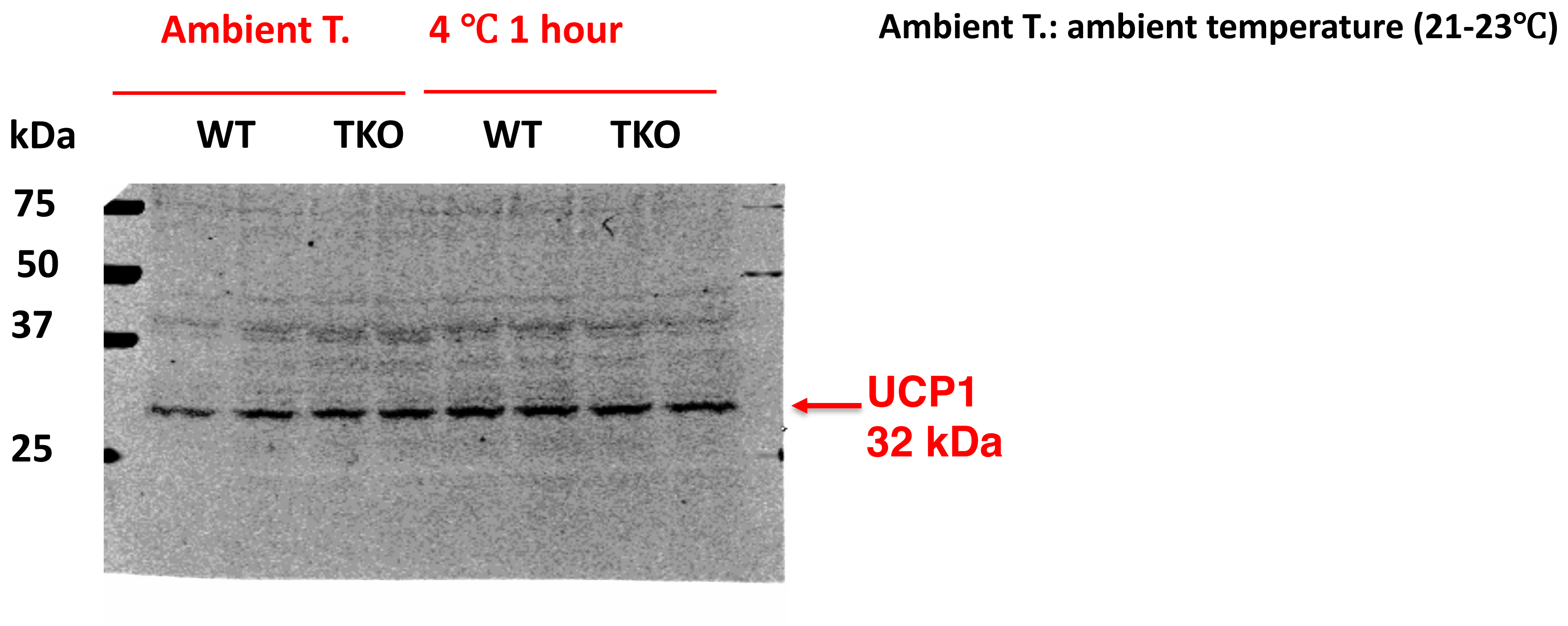

Figure 1-figure supplement 1-source data 2
Interscapular brown adipose tissues were collected from both ambient temperature housed and 4°C 1 hour exposed WT and TKO mice and 30 μg of the tissue lysate were used for UCP1 (33 kDa) immunoblotting analysis as described in the Immunoblotting, Method details. The raw image was used for Figure 1-figure supplement 1 B.

Figure 1-figure supplement 1-source data 3

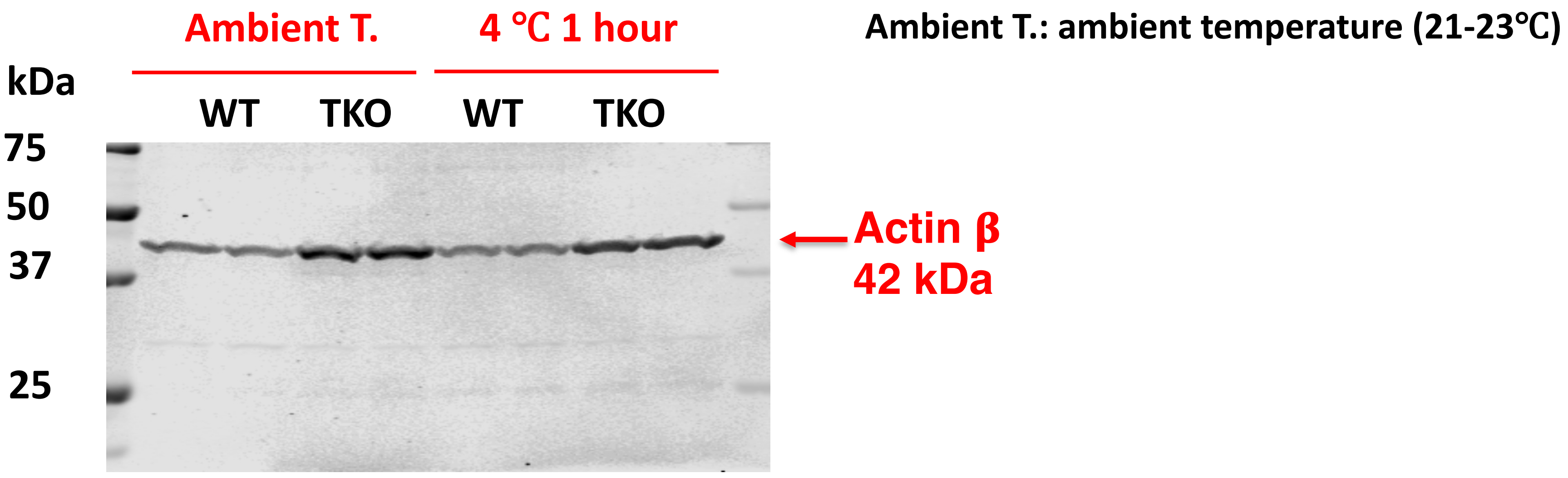

Figure 1-figure supplement 1-source data 3

Interscapular brown adipose tissues were collected from both ambient temperature housed and 4°C 1 hour exposed WT and TKO mice and 30 μg of the tissue lysate were used for actin β immunoblotting analysis as described in the Immunoblotting, Method details. The raw image was used for Figure 1-figure supplement 1 B.
